# Mutations in genes involved in nonsense mediated decay ameliorate the phenotype of *sel-12 *mutants with amber stop mutations in *Caenorhabditis elegans*

**DOI:** 10.1186/1471-2156-10-14

**Published:** 2009-03-20

**Authors:** Alisson M Gontijo, Sylvie Aubert, Ingele Roelens, Bernard Lakowski

**Affiliations:** 1 Nematode Genetics Group, Department of Neuroscience, Institut Pasteur, 25 rue du docteur Roux, Paris, France; 2 Current address : Instituto de Neurociencias, CSIC-UMH, Unidad de Neurobiología del Desarrollo, Campus de Sant Joan, Apto 18. 03550 Sant Joan d'Alacant, Alicante, Spain; 3 Current address : Unité postulante de Pathogenèse de Helicobacter, Institut Pasteur, 28 rue du docteur Roux, Paris, France; 4 Current address : Unité de Génétique Humaine et Fonctions Cognitives, Institut Pasteur, 25 rue du docteur Roux, Paris, France

## Abstract

**Background:**

Presenilin proteins are part of a complex of proteins that can cleave many type I transmembrane proteins, including Notch Receptors and the Amyloid Precursor Protein, in the middle of the transmembrane domain. Dominant mutations in the human presenilin genes PS1 and PS2 lead to Familial Alzheimer's disease. Mutations in the *Caenorhabditis elegans sel-12 *presenilin gene cause a highly penetrant egg-laying defect due to reduction of signalling through the *lin-12*/Notch receptor. Mutations in six *spr *genes (for suppressor of presenilin) are known to strongly suppress *sel-12*. Mutations in most strong *spr *genes suppress *sel-12 *by de-repressing the transcription of the largely functionally equivalent *hop-1 *presenilin gene. However, how mutations in the *spr-2 *gene suppress *sel-12 *is unknown.

**Results:**

We show that *spr-2 *mutations increase the levels of *sel-12 *transcripts with Premature translation Termination Codons (PTCs) in embryos and L1 larvae. mRNA transcripts from *sel-12 *alleles with PTCs undergo degradation by a process known as Nonsense Mediated Decay (NMD). However, *spr-2 *mutations do not appear to affect NMD. Mutations in the *smg *genes, which are required for NMD, can restore *sel-12(PTC) *transcript levels and ameliorate the phenotype of *sel-12 *mutants with amber PTCs. However, the phenotypic suppression of *sel-12 *by *smg *genes is nowhere near as strong as the effect of previously characterized *spr *mutations including *spr-2*. Consistent with this, we have identified only two mutations in *smg *genes among the more than 100 *spr *mutations recovered in genetic screens.

**Conclusion:**

*spr-2 *mutations do not suppress *sel-12 *by affecting NMD of *sel-12(PTC) *transcripts and appear to have a novel mechanism of suppression. The fact that mutations in *smg *genes can ameliorate the phenotype of *sel-12 *alleles with amber PTCs suggests that some read-through of *sel-12(amber) *alleles occurs in *smg *backgrounds.

## Background

Presenilin proteins are part of a complex of proteins that can cleave many type I transmembrane proteins with short extra-cellular domains [1]. Two of the best characterized substrates of the presenilin complex are the amyloid precursor protein (APP) and Notch-type receptors. Presenilin activity is necessary for the generation of β-amyloid, the major constituent of the senile plaques found post-mortem in the brains of patients who had suffered from Alzheimer's disease [1]. Mutations in APP, and in the two human presenilin genes PS1 and PS2, dominantly cause early onset familial Alzheimer's disease. Presenilins are also absolutely required for the signalling through Notch receptors and in all animals examined, the complete loss of presenilin activity is lethal due to developmental defects arising from the absence of Notch signalling.

In *Caenorhabditis elegans *there are three presenilin genes. Mutations in *spe-4 *lead to sterility and this gene appears to have a very specific role in spermatogenesis [2,3]. The two other presenilin genes, *sel-12 *and *hop-1*, each have broader, and partially redundant roles throughout the organism and are both required for proper Notch signalling [4-8]. In the absence of *hop-1*, animals have reduced fertility [5,6] while *sel-12 *mutants show a defect in egg-laying behaviour [4,7,8]. *hop-1; sel-12 *double mutants have a lethal phenotype and show defects in all known LIN-12 and GLP-1 signalling decisions [5,6]. The phenotype of *sel-12 *mutants is due to defects in two *lin-12*/Notch-dependent developmental decisions that occur in the mid/late larval stages. In *sel-12 *animals there is often a defect in determination of the π cell fate and in the development of the connection between the vulva and the uterus [7]. In these animals the passage way between the uterus and the vulva remains blocked by thick tissue, which everts in the early adult stage giving rise in the adult stage to a protruding vulva phenotype (Pvl). However, this Pvl phenotype, which can easily be seen in a dissecting microscope, is only partially penetrant and even in the strongest alleles only 3/4 of all animals display it [8]. *sel-12 *animals also display a highly penetrant defect in the orientation of the vulval muscles that open the vulva to allow egg-laying. The muscle defects vary from animal to animal, but in strong *sel-12 *alleles no animals show a completely normal pattern of sex muscle orientation and consequently, strong *sel-12 *alleles display a completely penetrant egg-laying (Egl) defect [8].

In order to find out more about the biological roles of presenilins, we and others have done several screens for suppressors of the egg-laying defect of *sel-12*. Six genes (*spr-1-spr-5 *and *sel-10*) have been identified that strongly suppress the *sel-12 *defects [9-14]. In the strongest suppressor mutations, the penetrance of *sel-12 *defects is reduced to less than 10% and in the *spr-3 *gene to almost 0% [13]. However, many *spr *mutations, especially those that suppress the *sel-12 *phenotype more weakly, remain uncharacterized.

Mutations in the strong *sel-12 *suppressor genes *spr-3, spr-4 *and *spr-5 *have been shown to de-repress the transcription of the *hop-1 *presenilin gene and probably suppress *sel-12 *by replacing the activity of one presenilin with another [11,13]. SPR-1, SPR-3, SPR-4 and SPR-5 resemble components of the mammalian REST-CoREST transcriptional repressor complex [11-13]. Furthermore, SPR-1 physically interacts with SPR-5 in GST pull down and in yeast two hybrid experiments [11]. This suggests that SPR-1 probably functions together with SPR-3, SPR-4 and SPR-5 to regulate *hop-1 *transcription [14]. An additional suppression mechanism is provided by *sel-10*. SEL-10 is an F-box protein that is part of an E3 ubiquitin ligase complex [15] that targets proteins for degradation by the proteosome. Mutations in *sel-10 *result in the stabilization of its target proteins which include the presenilins and the intracellular domain of Notch receptors [9,16-20]. Thus *sel-10 *probably suppresses *sel-12 *by either increasing HOP-1 protein levels, by increasing the half-life of LIN-12 intracellular fragment, or by both.

Although *spr-2 *was the first *spr *gene to be cloned, how it suppresses *sel-12 *on a molecular level remains unknown [10]. Unlike *sel-10 *mutations, mutations in *spr-2 *have no obvious effects on *lin-12 *signaling [10]. However, genetic evidence indicates that, like *spr-1, spr-3, spr-4 *and *spr-5*, *spr-2 *does not bypass the requirement for presenilin activity, because a *hop-1;spr-2;sel-12 *triple mutant is as unviable as is a *hop-1;sel-12 *double mutant [10]. SPR-2 is a Nucleosome Assembly Protein (NAP) orthologous to the human SET/Taf-1beta/INHAT oncogene [10]. SET has been implicated in a wide range of processes including facilitating transcription, transcriptional repression, inhibition of Protein Phosphatase 2A, regulation of the cell cycle and the membrane recruitment of Rac1 during cell migration [21-26]. However, apart from the fact that *spr-2 *mutations can suppress *sel-12*, all the *spr-2 *mutants isolated so far are viable and have no strong phenotypes [10].

Nonsense mediated decay (NMD) is a normal cellular process in eukaryotes in which messenger RNAs containing Premature translation Termination Codons (PTCs) are targeted for degradation [27-29]. In *C. elegans *seven *smg *(suppressor mutations with morphological effects on genitalia) genes were identified and found to be essential for NMD [30-32]. More recently, two additional genes that play a role in NMD were identified but reduction of function of these genes is lethal so they were given the names *smgl-1 *and *smgl-2 *(for *smg *and lethal) [33].

Cycles of phosphorylation and de-phosphorylation of the protein SMG-2, the ortholog of yeast UPF1 [34,35], plays a central role in the process of NMD [36]. In the nematode, SMG-1, SMG-3 and SMG-4 act to phosphorylate SMG-2, whereas *smg-5, smg-6 *and *smg-7 *are required for SMG-2 dephosphorylation. *smg-2 *encodes an ATPase/helicase that can bind to mRNAs containing PTCs with greater affinity than those without. This process is enhanced by the presence of SMG-3 and SMG-4 [37], which are known as UPF2 and UPF3, respectively, in yeast [38,39]. SMG-1, a conserved Phosphatidylinositol Kinase-Related Protein Kinase [36], then phosphorylates SMG-2 in a SMG-3- and SMG-4-dependent manner [40]. *smg-5, smg-6 *and *smg-7 *encode related proteins all containing an N-terminal 14-3-3 like domain [41], and are thought to recognize phosphorylated SMG-2 and trigger its dephosphorylation by recruiting protein phosphatase 2A (PP2A) into a SMG-5-PP2A sub-complex [42-44]. SMG5 and SMG6 proteins further share a C-terminal PINc domain (an RNAse H-related domain), although only SMG6 has conserved the canonical triad of acidic residues that are crucial in RNAse H for nuclease activity [45]. These three *smg *genes were initially thought to be absent from yeast, however, it has been recently determined that the metazoan SMG6 proteins are orthologous to yeast Ever shorter telomeres 1 (Est1a) [46-48]. Also, Ebs1, a second yeast gene with sequence similarity to Est1, shares functional and molecular similarities to hSMG5/7 (although similarities are greater with hSMG7) [49]. While both yeast Est1 and Ebs1 are involved in telomere maintenance, a role for *smg *genes in telomere maintenance has not yet been demonstrated in *C. elegans*. However, other NMD genes do play a role in telomere function in yeast [50] and in humans, where they play a negative role by regulating the association with chromatin of telomeric repeat-containing RNA and by protecting chromosome ends from telomere loss [51].

Here, we present experiments in which we tried to determine better how the strong suppressor gene *spr-2 *suppresses *sel-12*. We show that, although *spr-2 *does not affect *hop-1 *transcript levels, *spr-2 *mutations do increase *sel-12(PTC) *transcript levels in the embryo and in the L1 larvae. This study led us to examine the effects of *smg *mutations on *sel-12 *transcript levels and in modulating the *sel-12 *phenotype. We find that the transcripts of three *sel-12 *alleles with PTC mutations are subject to NMD and that *smg *mutations restore nearly normal *sel-12 *transcript levels. Furthermore, we show that a new weak suppressor mutation we had identified, *spr-8(pf52)*, is an allele of *smg-6 *and that another mutation identified in a putative weak *spr *strain, *by146*, is an allele of *smg-1*. *smg *mutations can weakly ameliorate the phenotype of *sel-12 *alleles with amber stop mutations but do not affect the phenotypes of missense, deletion or opal stop alleles. This suggests that some read through of *sel-12(amber) *alleles occurs in *smg *backgrounds. Furthermore, we show that *spr-2 *does not appear to be affecting NMD and that the suppression of *sel-12 *by *spr-2 *can not be explained by the effect of *spr-2 *mutations on *sel-12(amber) *transcript levels. Our results suggest that *spr-2 *suppresses *sel-12 *by a novel mechanism.

## Results

### Presenilin transcript levels in *spr-2 *mutants

**Figure 1:**
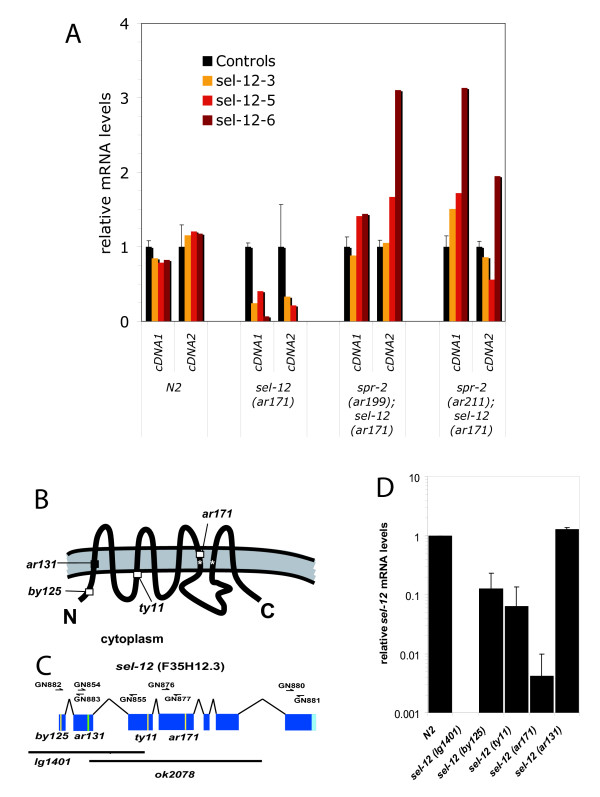
**The effect of *spr-2 *and *sel-12 *mutations on *sel-12 *transcript levels**. A) mRNA levels determined by qRT-PCR relative to the average of control transcripts (*ama-1, nhx-4 *and *eft-2*). *sel-12 *transcripts were detected from synchronized L1s. Three different amplicons, *sel-12-3, sel-12-5 *and *sel-12-6*, (see Table 1 for primer names and sequences) were tested. The error bars represent the standard deviation between the normalized control primers. *sel-12 *transcript levels are greatly reduced by the *ar171 *mutation, while both *spr-2 *mutations reproducibly increase *ar171 *transcript levels. B) A model of the SEL-12 presenilin protein where the position of the point mutations are depicted. Stop mutations are shown by open squares and amino acid substitutions by black squares (Modified from [6]). C) The gene structure of *sel-12*(F35H12.3). Exons are denoted by blue rectangles joined by a jointed line. The 3' UTR of *sel-12 *is noted in lighter blue. The position of alleles PTC mutations are indicated by yellow vertical bars, the *ar131 *missense mutation is indicated with a green vertical bar and the extent of the *ok2078 *and *lg1401 *deletions is indicated with black horizontal bars. The position of the primer pairs used in qRT-PCR studies presented in this paper are indicated with small arrows above the gene structure. D) The relative mRNA levels of *sel-12 *detected by qRT-PCR with the primer pair for amplicon *sel-12-6 *(Table 1) are plotted on a logarithmic scale for N2, and strains with five different *sel-12 *mutations.

In order to determine if *spr-2 *functions through a similar mechanism to other *spr *genes to suppress *sel-12*, we looked at the effect of mutations in *spr-2 *on presenilin transcript levels. We find that *spr-2 *mutations do not increase the amounts of *hop-1 *or *spe-4 *transcripts (Gontijo and Lakowski unpublished data). However, *spr-2 *mutations increase the levels of *sel-12(ar171) *mutant transcripts in the L1 stage (Figure 1A). The *sel-12 *allele tested, *ar171*, contains a Premature translation Termination Codon (PTC) that is predicted to truncate the SEL-12 protein before the large cytoplasmic loop region (See Figure 1B for scheme). Transcripts containing a PTC are known to be targeted for degradation in a translation dependent manner by the nonsense mediated decay (NMD) machinery [52].

### *sel-12(PTC) *transcripts are subject to NMD

So we looked at the transcript levels in the wild type and in five *sel-12 *alleles: three, *ar171, ty11*, and *by125 *containing PTC mutations, one missense allele, *ar131*, and a deletion, *lg1401 *(Figure 1B, C). The *lg1401 *deletion eliminates the annealing sites of the primers used to detect the *sel-12 *transcript (Figure 1C and Table 1) so in this strain *sel-12 *is undetectable (Figure 1D). In all three of the PTC alleles, the transcript levels are greatly reduced (Figure 1A and 1D and Additional file 1). On the other hand, the missense *ar131 *mutation has transcript levels comparable to the wild type (Figure 1D). This clearly shows that PTC mutations in *sel-12 *are subject to NMD. *sel-12 *is the upstream gene in an operon with F35D12.5 but *sel-12(PTC) *mutations do not strongly affect the transcript levels of F35H12.5 (data not shown).

**Table 1:** PCR primers used in this study

Product	Sense primer	Antisense primer	enzyme
*Rpl-12*	GN1100 GTTGCGTCGGAGGAGAAGTCG	GN1101 GATGATGTCGTGTGGGTGTTGTC	
*ama-1*	GN701 GTCACATGAAAGATGGCGATATAA	GN702 CATCATTGACTTTTGTGGAGAGT	
*Eft-2*	GN739 ACTTGTTGGAGTCGATCAATACCT	GN740 GACTGGAGATACGGAGAACTTCAT	
*nhx-4*	GN753 GGGTTGTACCATTAAGTTCGTCAT	GN754 TACTTGTCGTTCAGGTGAGTAAGC	
*sel-12-1*	GN854 GGAGCATCTCACGTTATTCATCTA	GN855 CCCGGACAAAAGGAGTGTATAGTA	
*sel-12-3*	GN876 TGGACTGGGTAACTATGGAGTTCT	GN877 GATAAAGACCAGAGCCATTAGTGC	
*sel-12-5*	GN880 AGAGAGAGGTGTGAAACTTGGTCT	GN881 AGTGAAGCAGAGACCGATAAGAAT	
*sel-12-6*	GN882 CTTCCACAAGGAGACAACAGG	GN883 ATAACGTGAGATGCTCCGTATTTC	
Y22D7AL[4]	GN37 CTTACGCCAAGGACGTCAAG	GN38 GGTATTTGTCCTTGAGGTCG	DraI
*pkP3086*	GN553 TTGAGAAGAATGAGCAGAGCGG	GN554 AACTGATGGCCTAGAAATCCAAGGAGTTCG	DdeI
*Y71H2B*[2]	GN39 AAGAAGAATTAGGCGATGCGG	GN40 CTGAAAACTTGGAAAATCGGTG	DraI
C48B6[1]	GN124 GTCAAGGACCATTGTTACGAG	GN125 GGGCAGTTATTAGTTCGTGAG	DraI
*byP4*	GN1008 GAAATTTCAGTGCCACTTCC	GN1009 CTAGGCAGTACATAAATGCG	KpnI
*smg-1(by146)*	GN1222 CTTCATCTTGATGAACGAGTCATGC	GN1213 AGAAGTAGAACGGACTATGACACG	
*smg-1(by146)*	GN1222 CTTCATCTTGATGAACGAGTCATGC	GN1264 CATGATCTGCGAATGCTAGACG	

Primers used for quantitative PCR, the *rpl-12 *test and Single Nucleotide Polymorphism (SNP) mapping are noted. For SNPs, the restriction enzyme used to detect the polymorphism in indicated in the last column.

### The effect of *spr-2 *on *sel-12(PTC) *transcripts is stage specific

**Figure 2:**
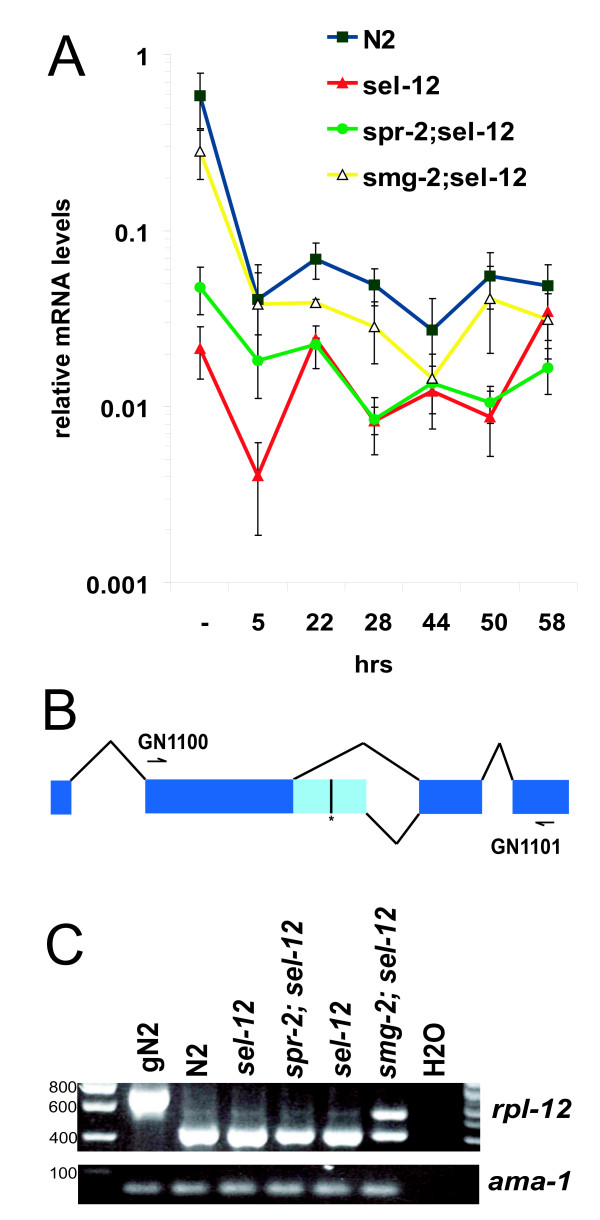
**The effect of *spr-2 *on *sel-12 *and *rpl-12 *transcripts levels**. A) The expression of *sel-12 *transcripts during development in: N2, LA242 *sel-12(ty11)*, LA259 *spr-2(ar199); sel-12(ty11) *and *smg-2(e2008); sel-12(ty11) *strains. The relative mRNA levels were determined by qRT-PCR with primer pairs for amplicon *sel-12-6 *(Table 1). The X axis represents time in hours after feeding synchronous starved L1s as described in the Methods section. (-) is asynchronous eggs. B) A scheme of the alternative splicing of *rpl-12*. Coding exons are shown in dark blue, the alternative 3' extension of exon 2 is shown in light blue with a bar and a * to indicate a stop codon. The locations of the primers used to detect *rpl-12 *transcripts are noted. C)*rpl-12 *mRNA splicing assay for Smg activity. RNA was prepared from synchronous L1s. Pictures are from Ethidium bromide-stained 2% agarose gel. The sizes of DNA fragments from the loading ladder are shown at the left. The gN2 lane is a control with N2 genomic DNA.

The initial observation of mutant *sel-12 *mRNA stabilization by *spr-2 *mutations was made in synchronous L1 *sel-12*(*ar171) *animals (Figure 1A and data not shown). To examine the effect of *spr-2 *on *sel-12 *transcript levels more thoroughly, we determined the *sel-12 *transcript levels in the wild type (N2), LA242 *sel-12(ty11)*, LA259 *spr-2(ar199); sel-12(ty11) *and *smg-2(e2008); sel-12(ty11) *strains at different times during development (Figure 2A). *sel-12 *is expressed strongly in all stages but most strongly in eggs. These results are similar to what we have seen by Northern blot [13] when we correct for the fact that the control in the Northern blot, *ama-1*, is expressed at higher levels in the eggs. The *ty11 *mutation greatly reduces *sel-12 *transcript levels at all developmental stages. *smg-2 *mutations greatly increases *sel-12(ty11) *levels close to those of the wild type, except at 44 hours. However, the effect of the *spr-2 *mutation on the *ty11 *transcript is much more modest and essentially limited to the eggs and L1 stage.

### *spr-2 *mutations do not affect NMD more generally

To see if *spr-2 *mutations had a more general effect on RNA levels, we tested whether *spr-2 *could influence NMD in several ways. Firstly, we made an *unc-54(r293); spr-2(ar199) *double-mutant strain. *unc-54 *codes for a major muscle myosin. In strains containing the *r293 *allele, the *unc-54 *transcript is unstable and subject to NMD, yielding paralyzed adult animals. Mutations in any *smg *gene stabilize *unc-54(r293) *transcripts so that the animals can move more normally [30]. *unc-54;spr-2 *double mutants are still strongly paralyzed, suggesting that *spr-2 *is not a *smg *gene.

The ribosomal protein gene, *rpl-12*, is normally alternatively spliced to produce two isoforms [53]. One form is unproductively spliced and contains a piece of an intron with a Premature translation Termination Codon (PTC) and another productively spliced, containing no intronic sequences and no PTC (Figure 2B). Although the *rpl-12 *PTC transcript is constitutively produced, it is actively degraded by NMD, so that its levels are normally very low with respect to the other isoform. In the absence of a *smg *gene, however, the *rpl-12 *PTC transcript accumulates and can be readily detected by reverse transcriptase PCR (RT-PCR), yielding a robust test for Smg activity [53].

So we prepared mixed stage RNA from a *spr-2 *homozygous strain and tested whether there would be any detectable enrichment of the *rpl-12 *PTC isoform, but found none (Figure 2C), confirming that *spr-2 *mutants are proficient in NMD. As the effect of *spr-2 *on *sel-12 *transcripts was found only in early stages, it could be that *spr-2 *affects NMD only in these particular stages of development. However, NMD of *rpl-12 *happens normally in a *spr-2(ar199) *strain at all the stages in which we have looked, even in embryos and L1s (data not shown), suggesting no general role for *spr-2 *in NMD at any developmental stage.

### *smg *mutations ameliorate the phenotype of *sel-12(ty11)*

**Figure 3:**
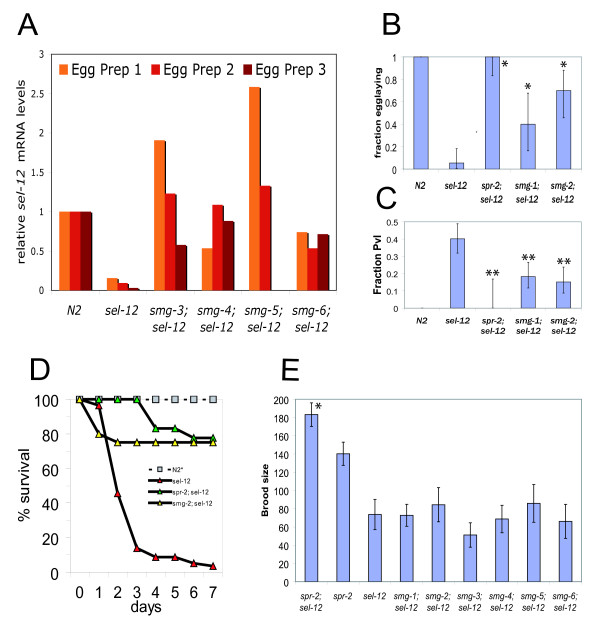
***smg *mutations ameliorate the phenotype of *sel-12(ty11)***. A) qRT-PCR analysis of *sel-12 *mRNA (amplicon *sel-12-6*) from asynchronous eggs from different genotypes. Results are shown for three independent Egg preparations, except for *smg-5; sel-12*, where only two biological repeats were performed. By a paired sample one-tailed Student's T-test, all four *smg *mutations significantly increase *sel-12 *transcript levels. B) Phenotypic quantification of the fraction of animals laying eggs (see Additional file 2, expt. 1 for the data) and C) the fraction of animals with a pronounced (*sel-12*-like) protruding vulva (Pvl); 55/137, 0/20, 21/115 and 15/99 for LA242 *sel-12(ty11)*, LA259 *spr-2(ar199); sel-12(ty11)*, LA613 *smg-1(r861); sel-12(ty11) *and *smg-2(e2008); sel-12(ty11) *animals respectively ± 95% confidence limits. Values statistically significantly higher than *sel-12(ty11) *are indicated with a * and those values significantly lower than *sel-12(ty11) *are indicated with **. Many N2 animals were observed but none showed a strong Pvl phenotype and all laid eggs. D) Survival curve of adult worms. At least 20 worms of each genotype were singled as L3s and checked daily for death. E) Total brood size quantification of a panel of mutants ± the 95% confidence limits of the mean. Values statistically significantly higher than *sel-12(ty11) *are indicated with a *. Results are also tabulated in Additional file 2 experiment 1.

In the process of doing these experiments we discovered that a *smg-2 *mutation partially ameliorates the phenotype of *sel-12(ty11)*. To see if other *smg *genes could also ameliorate the *sel-12(ty11) *phenotype, we made additional *smg; sel-12(ty11) *double mutants. Indeed, mutations in all *smg *genes that we tested greatly increased the levels of *sel-12(ty11) *transcripts (Figure 3A). Most *smg; sel-12(ty11) *double mutants also appeared to have some amelioration of the *sel-12 *phenotype. The ability of *sel-12 *animals to lay eggs can be scored in different ways which we quantified in both a *smg-1 *and a *smg-2 *background. More *smg; sel-12(ty11) *double mutants can lay some eggs than *sel-12(ty11) *animals (Figure 3B). Furthermore, fewer *smg; sel-12(ty11) *animals display a strong protruding vulva phenotype (Figure 3C), even though *smg *mutations often affect vulval morphology. Finally, most *sel-12 *animals die during the reproductive period due to internal hatching of progeny. These progeny develop further in the mother, feeding off her tissue, leading to a terminal 'bag-of-worms' phenotype where the developing larvae are encased by the empty cuticle of the mother. The internal hatching of progeny severely limits the lifespan of animals. However, in *smg; sel-12(ty11) *animals, many animals survive the reproductive period (Figure 3D). In *smg; sel-12(ty11) *animals, the restoration of egg-laying behavior is variable and animals can display severe side-effects seldom seen in *spr; sel-12(ty11) *or *sel-12(ty11) *mutants alone, such as exploding during the L4 to adult molt and increased sterility (data not shown). Although animals with these problems were excluded from our analysis, we found that these *smg *mutations do not obviously increase brood size (Figure 3E). The brood size remained very similar to that of *sel-12 *with only very modest increases in a few of the *smg; sel-12(ty11) *double mutants that were not statistically significant (Additional file 2, Expt.1). The phenotypes of *smg; sel-12(ty11) *strains clearly contrasts with that of the LA259 *spr-2(ar199) IV; sel-12(ty11) *strain. For all phenotypes examined, *smg *mutations suppress *sel-12(ty11) *much more weakly than *spr-2(ar199) *mutation (Figures 3B, C and 3E).

### Isolation and characterization of *smg-6(pf52)*

**Figure 4:**
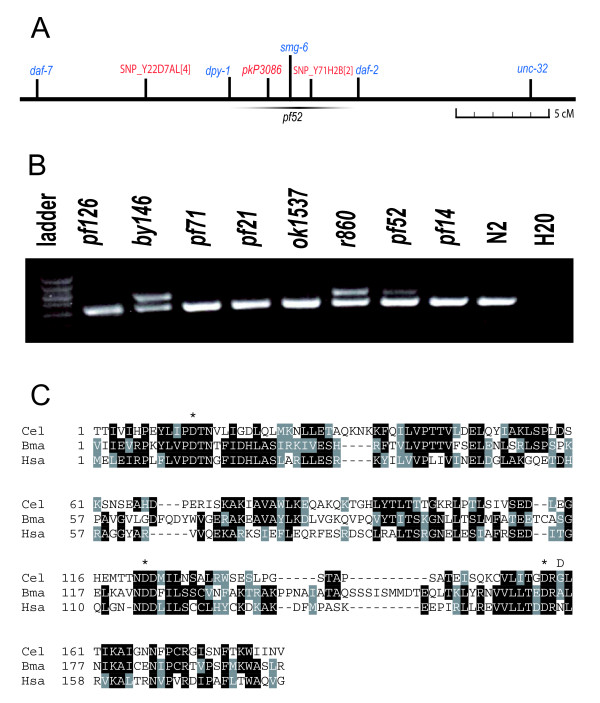
**The *spr *alleles *pf52 *and *by146 *affect NMD**. A) The genetic position of *spr-8(pf52)*. The genetic position of *pf52 *is shown with the position of classical genetic markers in blue and Single Nucleotide Polymorphisms (SNPs) in red used to position *pf52*. *smg-6 *is also found within the minimal region for *spr-8 *B) The results of the *rpl-12 *test for several strains. A 2% agarose gel with PCR fragments from *rpl-12 *is shown. N2 controls only have the smaller product while *smg-5(r860) *animals show both. The weak *spr *alleles *pf52 *and *by146 *have defects in NMD, while *pf14*, *pf21*, *pf71, pf126 *and *ok1537 *have retained NMD. C) An alignment of the PINc domain of SMG-6 from *Homo sapiens *(Hsa accession # NP_060045), *Brugia malayi *(Bma accession # EDP30730.1) and *Caenorhabditis elegans *(Cel accession # NP_497566.3). The sequences were aligned by ClustalW and analyzed by Boxshade. Identical amino acids are shaded in black while similar amino acids are shaded in grey. The three catalytic aspartates are denoted by a * above the sequence. The position of the *pf52 *G→D mutation is shown by a D above the sequence just after the last catalytic aspartate.

In our *spr *screens we have recovered many weaker mutations that allow some *sel-12 *animals to start laying eggs as young adults. However, most of these animals eventually become Egl and die from internal hatching of progeny. In the process of doing the studies on the effect of *smg *genes on *sel-12 *phenotype, we realized that one of the weak *sel-12 *suppressors that we had been trying to positionally clone mapped near *smg-6*. The *pf52 *allele complemented all of the known *sel-12 *suppressor genes, so we tried to map it genetically. *pf52 *is linked to the left arm of LGIII, where no other *spr *genes have been mapped. So we gave *pf52 *the gene name *spr-8*. The *spr-8(pf52) *mutation was mapped by a combination of classical and Single Nucleotide Polymorphism (SNP) mapping (see Methods) about mid way between *dpy-1 *and *daf-2*. The SNP mapping data was most consistent with a position between the SNPs *pkP3086 *and SNP_Y71H2B[2] (Figure 4A). The gene *smg-6*, which is involved in nonsense mediated decay (NMD), maps to the same genetic interval as *spr-8 *(Figure 4A). The *pf52 *mutation was recovered as a suppressor of the amber stop allele *ty11*. So we tested whether *pf52 *could be allelic to *smg-6*. We tested complementation of *pf52 *to *smg-6(r896) *for *sel-12(ty11) *suppression and found that some *spr-8(pf52)/smg-6(r896); sel-12(ty11) *animals were clearly suppressed in the first heterozygous generation and their progeny appeared to lay more eggs than *sel-12(ty11)*. Moreover, *pf52 *strongly suppresses the movement defects of *unc-54(r293) *animals and displays a partial maternal effect for *unc-54 *suppression (See Methods). Consistent with this, *smg-6 *is known to have a maternal effect [30,54]. Finally, the PTC form of *rpl-12 *clearly accumulates in *pf52 *strains but that *pf52 *has a weaker defect in NMD than most other *smg *genes (Figure 4B and data not shown). Interestingly, *smg-6(r896) *is known not to completely abolish NMD [54], and also shows this same weak effect on *rpl-12 *PTC transcript accumulation (data not shown).

*smg-6 *corresponds to the predicted gene Y54F10AL.2 (Philip Anderson personal communication). There are three predicted isoforms of *smg-6 *so we sequenced all coding exons in the longest form of the gene (Y54F10AL.2A) and found that the *pf52 *has a G → A mutation at position 3660 of the transcript [GenBank:NM_065165] that leads to an G → D amino acid substitution at position 1216 in the protein [GenBank:NP_497566.3]. *smg-6 *has also been named *est-1 *as it is similar to the *Saccharomyces cerevisae *gene Ever Shorter Telomeres-1. An alignment of the PINc domain of SMG-6 proteins from the Human, and the nematode species *Brugia malayi *and *C. elegans *are shown in Figure 4C. The *smg-6(pf52) *mutation affects an amino acid very close to the final aspartate of the putative catalytic domain.

### Isolation and characterization of *smg-1(by146)*

To test whether any other mutations we recovered in the different *spr *screens were additional mutations in *smg *genes, we prepared mixed stage RNA from the original isolate strain of all previously uncharacterized *spr *mutations and tested the effects of these mutations on the stability of the *rpl-12 *PTC isoform. All of these strains, except one, show no effect on the stability of the long form of *rpl-12 *(Figure 4B and data not shown). However, the BR2097 *by146; sel-12(ar171) unc-1(e538) X *strain has high levels of the *rpl-12 *PTC isoform that are comparable to that seen with the strongest *smg *mutations we have tested and much stronger than *smg-6(pf52) *(Figure 4B and data not shown).

To confirm that BR2097 contains a *smg *mutation, we out-crossed BR2097 and could recover weakly Pvl non-Unc strain that did not contain either *sel-12(ar171) *or *unc-1(e538)*. All other *smg *mutations have been shown to have mild vulva defects. *by146 *can completely suppress the paralyzed phenotype of *unc-54(r293)*, confirming that it is a *smg *mutation. *by146 *is tightly linked to SNP_C48B6[1] and *byP4 *(see Table 1), two SNPs in the cluster of LG I (data not shown) close to *smg-1, smg-*5 and *smgl-1*. No mutations have been isolated in *smgl-1 *so we tested complementation of *smg-1(r861) *and *smg-5(r860) *to *by146*. We found that *by146 *complements *smg-5(r860)*, but fails to complement *smg-1(r861) *for the Pvl phenotype and for suppression of *unc-54(r293)*.

**Figure 5:**
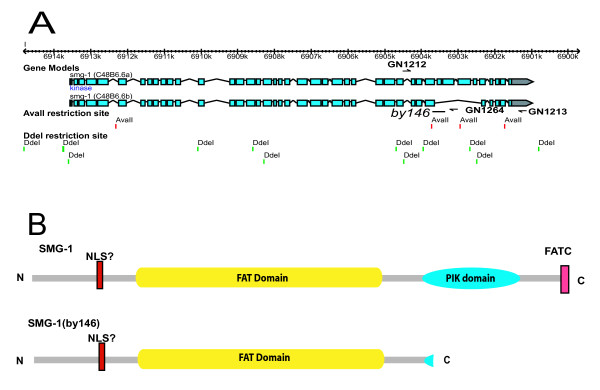
**The nature of the *smg-1(by146) *mutation**. A) The structure of the *smg-1 *gene with the position of the primers, GN1212, GN1213 and GN1264 used to detect and genotype the *by146 *mutation. The gene structure was captured from Wormbase  Wormbase release WS187). Below the gene structure the position of *AvaII *and *DdeI *restriction sites are shown by vertical bars along with the approximate position of the *by146 *mutation by a horizontal bar. B) The domains within the SMG-1 protein according to [40] along with the expected affect of the *by146 *mutation on the protein. NLS = Nuclear Localization Signal, FAT = FRAP, ATM, and TRRAP domain, PIK domain = phosphatidylinositol kinase (PIK) domain and FATC = FAT carboxyl terminus domain.

As the *by146 *mutation was recovered in a UV/TMP mutagenesis, we thought that there would be a good possibility that this allele would have a chromosomal rearrangement, such as a deletion, that could be detected by PCR. So we amplified the *smg-1 *genomic region in fragments of about 2–3 kb in both an N2 and *by146 *background and subjected these fragments to restriction digest with different enzymes. We determined that the *by146 *mutation had a small deletion in the 787 bp *AvaII *fragment (Figure 5A). So we sequenced this fragment and determined that *by146 *has a simple 190 bp deletion. The *by146 *deletion has the following breakpoints which are denoted by a slash:

GTGCTACACC/aatgttccac ... cccagcaaca/TGTATCACAA, where retained sequences are shown in capitals and deleted sequences in lower case. The *smg-1(by146) *deletion changes the reading frame and truncates the predicted SMG-1 protein so that it lacks the C-terminal 1/4 of the protein including most of the PIP3 kinase domain (Figure 5B). The PIP3 kinase domain is essential for SMG-2 phosphorylation and thus for NMD. Consequently, *by146 *is likely to be a strong loss of function or null allele of *smg-1*, consistent with its strong effect on the *rpl-12(PTC) *transcript (Figure 4B).

### *smg *mutations only suppress *sel-12(amber) *alleles

**Figure 6:**
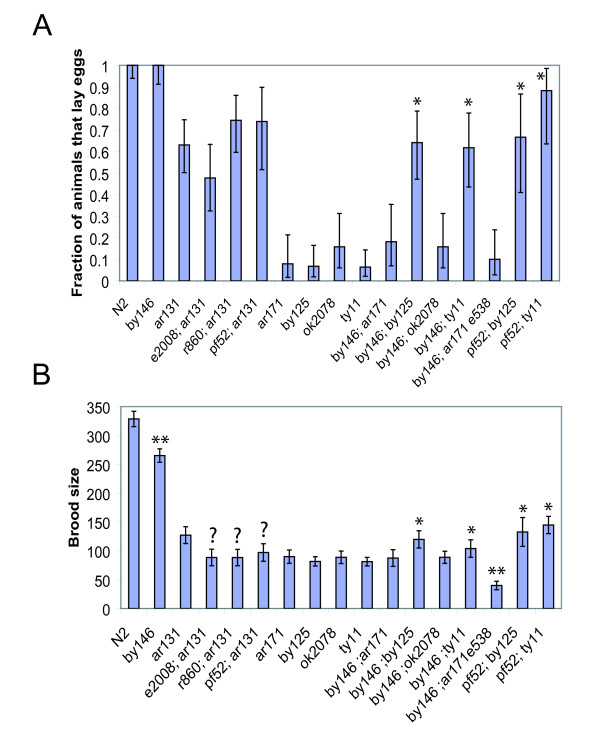
***smg-1(by146) *and *smg-6(pf52) *ameliorate the phenotype of *sel-12(amber) *mutations**. A) The fraction of animals that can lay some eggs ± the 95% confidence limits for the fraction for various *smg; sel-12 *strains and their controls. Values statistically significantly higher than the appropriate *sel-12 *control strain are indicated with a *. The number of animals that can lay some eggs is a very sensitive indicator of weak *sel-12 *suppression; however, most of the animals containing *sel-12 *mutations later became Egl. B) The mean brood size ± the 95% confidence of the mean for the strains examined in A. Values statistically significantly higher than the appropriate *sel-12 *control strain are indicated with a * and those values significantly lower than the appropriate control strain are indicated with **. *smg; sel-12(ar131) *strains may have a lower brood size than *sel-12(ar131) *as indicated by ?. Both *smg-1(by146) *and *smg-6(pf52) *increase the brood sizes of *sel-12(amber) *alleles but not that of *sel-12 *missense, deletion or opal alleles.

To test whether the effect of *smg *genes on the *sel-12 *phenotype was only through the stabilization of *sel-12(PTC) *transcripts, we tested whether *smg-2 *affected *hop-1 *or *spe-4 *mRNA stability, but we found that *smg-2 *had little or no effect on *hop-1 *or *spe*-4 mRNA levels at any developmental stage (data not shown). If *smg *mutations suppress *sel-12 *by stabilizing *sel-12(PTC) *transcripts then they should only suppress *sel-12(PTC) *alleles. So we compared the effects of placing *smg *mutations into the background of six different *sel-12 *alleles. *ty11 *and *by125 *are amber PTC alleles, *ar171 *is an opal PTC allele, *ok2078 *and *lg1401 *deletions that delete a large part of the *sel-12 *gene and should be null alleles and *ar131 *is an C60S amino acid substitution with a partial loss of function phenotype (see Figure 1C). In one experiment we directly compared the effects of *smg-6(pf52) *on *sel-12(by125) *and *sel-12(ty11) *alleles. Consistent with our hypothesis, *pf52 *equally ameliorated the proportion of animals that lay eggs as well as increasing average brood size of *sel-12(by125) *and *sel-12(ty11) *alleles (Figure 6 and Additional file 2 experiment 3).

In another experiment we found that *smg-2(e2008), smg-5(r860) *and *smg-6(pf52) *mutations do not ameliorate the *sel-12(ar131) *phenotype. *smg; sel-12(ar131) *animals have very similar Egl phenotypes to *ar131 *animals (Figures 6A and Additional file 2 experiment 2). Smg mutations do not increase the brood size of *sel-12(ar131) *animals and indeed actually might slightly reduce the number of progeny of *sel-12(ar131) *animals. The strains LA726 *smg-2(e2008)*; *sel-12(ar131) *and LA722 *smg-5(r860); sel-12(ar131) *had statistically lower brood sizes than the LA728 *sel-12(ar131) *control strain (Additional file 2 experiment 2). However, the LA725 *smg-2(e2008)*; *sel-12(ar131) *and LA723 *smg-5(r860); sel-12(ar131) *strains had brood sizes that were not statistically different from the LA728 *sel-12(ar131) *control strain (Additional file 2 experiment 2), indicating that the reduced brood sizes are not simply caused by the presence of the *smg *mutation. Additionally, Student's T-tests indicate that the brood sizes of the LA726 and LA725, as well as the LA723 and LA722 strains are not statistically different. Furthermore, when the results of LA722 and LA723 strains are pooled and when the results of the LA726 and LA725 strains are pooled, the brood size of *smg-2(e2008); sel-12(ar131) *and *smg-5(r860); sel-12(ar131) *animals does not statistically differ from that of LA728 *sel-12(ar131) *(P = 0.100 and P = 0.103 respectively, Student's T-test).

Similarly we found that *smg-5(r860); him-8(e1489); sel-12(lg1401) *hermaphrodites were as Egl and Pvl as those in a control *him-8(e1489); sel-12(lg1401) *strain. Finally we directly compared the effects of the *smg-1(by146) *mutation on the *sel-12 *alleles: *ar171, by125, ok2078 *and *ty11*. In two experiments, we see that *by146 *ameliorates both the egg laying phenotype and the brood size of *by125 *and *ty11 *(Figures 6A, B and Additional file 2 experiments 5 and 6). However, *by146 *has no consistent effect on the phenotype of *ok2078 *and *ar171 *(Figures 6A, B and Additional file 2 experiments 5 and 6). Thus our results show that *smg *mutations do not ameliorate the phenotype of *sel-12 *missense and deletion alleles as expected. Surprisingly, *smg *mutations ameliorate the phenotypes of amber PTC *sel-12 *alleles but not that of the *ar171 *opal PTC mutation.

## Discussion

### All *smg *genes affect *sel-12 *similarly

In the course of this study we have shown that *sel-12 *transcripts with premature termination codons are subject to NMD. The loss of nonsense mediated decay can restore *sel-12(PTC) *transcripts to near wild type levels. The different *smg *genes have different roles in mediating NMD. In particular, *smg-1, smg-3 *and *smg-4 *help to promote SMG-2 phosphorylation, while *smg-5, smg-6 *and *smg-7 *promote SMG-2 de-phosphorylation. Furthermore, *smg-2, smg-5 *and *smg-6 *have been implicated in the maintenance of RNAi while *smg-1 *is not [55]. However, in our initial study (Figure 3), we found no strong differences in the effects of the different *smg *genes on *sel-12(PTC) *transcript levels and *sel-12 *suppression. This suggests that *smg *mutations ameliorate the *sel-12(ty11) *phenotype simply by stabilizing *sel-12(ty11) *transcripts.

### *smg *mutations poorly suppress *sel-12*

Genes involved in NMD weakly ameliorate the phenotype of *sel-12(amber) *mutations. However, this effect is much weaker than the effect of mutations in strong *spr *genes such as *spr-2*. In the *spr *screens, we have identified only two *smg *mutations as weak suppressors of *sel-12 *in over 100 *spr *alleles indicating that screening for *sel-12 *suppression is an inefficient way to recover *smg *alleles. Although *smg *mutations weakly suppress *sel-12*, most *smg; sel-12(PTC) *animals still become Egl and die of internal hatching of progeny. Thus at any time, only a proportion of *smg; sel-12 *animals lay eggs and can be isolated for this phenotype. This may explain the relative paucity of *smg *alleles recovered in screens for suppressors of *sel-12(ty11)*. Furthermore, the absence of clear effects of *smg-1(by146) *on the phenotype of *sel-12(ar171) *suggests that *smg *should only be very poorly selected for in *ar171 *suppressor screens.

### The *by146 *mutation was recovered serendipitously

Curiously, the *by146 *allele was isolated in the BR2097 *by146; sel-12(ar171) unc-1(e538) *strain. However, our results show that *by146 *does not obviously suppress *sel-12(ar171)*, so it is unclear why it was recovered in a *sel-12(ar171) *background. The BR2097 strain was frozen soon after it was isolated and was not examined further until this study. Not all of our putative weak suppressor alleles have been characterised carefully, so we re-examined the phenotype of BR2097. BR2097 animals have a similar proportion of egg laying animals to all of the *sel-12 *null alleles including *ar171 *(Figures 6A and Additional file 2 experiment 6). Furthermore, the brood size of BR2097 is not elevated and is in fact significantly smaller than *ar171 *itself (Figures 6B and Additional file 2 experiment 6), an effect that might be due to the *unc-1(e538) *background mutation. These results indicate that the BR2097 strain does not appear to have a suppressor mutation that can suppress *sel-12(ar171) *and that the presence of the *smg-1(by146) *mutation could not have been selected for. This suggests that the isolation of *by146 *could have been due to the fortuitous presence of a background *smg-1 *mutation in the BR2097 strain that did not strongly affect the Egg-laying defect of *sel-12(ar171)*. However, our subsequent studies indicate that *smg-1(by146) *can ameliorate the phenotype of *sel-12(ty11) *and *sel-12(by125)*. Thus all of our results are consistent with *smg-1(by146) *being a weak amber allele specific suppressor of *sel-12*. We note that our results conflict with those of Levitan *et al. *who saw that *sel-12(ar171) *is somewhat suppressed by *smg-1(r861) *[56]. However, no data was provided to support this claim and this may highlight the necessity of quantifying the suppressor phenotype of weak *spr *mutations.

### The *pf52 *mutation suggests an important function for the PINc domain of SMG-6

The fact that *pf52 *affects the PINc domain of SMG-6 indicates that this domain has an important function in NMD. The highly conserved PINc domain of SMG-6 proteins was predicted to function as an RNAse [45]. Recently, it has been demonstrated that both in Drosophila and Human cell culture, SMG6 proteins act as RNAses that cleave mRNAs with PTCs near the PTC [57,58]. Furthermore, this activity is dependent on the PINc domain and the three catalytic aspartate residues in this domain [57,58]. There are also several basic residues in the PINc domain that may bind to RNAs [45]. The addition of an acidic amino acid in this region may destabilize RNA binding. So the *pf52 *mutation could affect the putative RNAse activity of SMG-6 by either disrupting the catalytic core, by affecting RNA binding, or both. The fact that *pf52 *does not eliminate NMD as well as alleles of other genes, including *smg-1(by146) *(see Figure 4B) suggests that either *smg-6 *is not completely required for NMD in *C. elegans*, or that alternately, the *pf52 *allele may be a partial loss of function mutation. However, in a non-complementation screen for *smg-6 *alleles several alleles were recovered including three homozygous lethal mutations [54]. This suggests that the null phenotype of *smg-6 *may be lethal and that *pf52 *does not completely eliminate *smg-6 *function.

### Smg mutations appear to affect fertility

Although they were not directly compared, the suppression of *sel-12(amber) *mutations by our alleles, *smg-1(by146) *and *smg-6(pf52) *was clearer, especially for brood size (see Figure 6B and Additional file 2 experiments 5 and 6), than with reference alleles ordered from the Caenorhabditis Genetics Center (CGC) (Figure 3E). Indeed, in many *smg; sel-12(ty11) *strains with *smg *alleles from the CGC collection we saw a high degree of sterility. The sterility in the *smg-2(e2008); sel-12(ty11) *strain was so high that we were not able to continue propagating the strain and it was subsequently lost. Our alleles have been propagated for a limited number of generations while the reference alleles of the *smg *genes were isolated over 20 years ago [30]. Our data also suggest that *pf52*, the allele with a weak effect on NMD (Figure 4B and Additional file 1), may suppress *sel-12 *better than the *by146 *allele which has completely lost NMD (Figures 4B, 6B and Additional file 2). All of this suggests that there might be negative effects on fertility of eliminating NMD and maintaining strains without NMD for many generations. Consistent with this, our results show that LA920 *smg-1(by146) *has a clearly reduced brood size as compared to N2 (Figure 6B and Additional file 2). Additionally, when the LA920 *smg-1(by146) *strain was cultured at the stressful temperature of 25°C for several generations, we saw that some animals had greatly reduced number of progeny (Lakowski data not shown), suggesting that some epigenetic effect may underlie the severely reduced fertility seen in some *smg; sel-12 *animals. As genes involved in NMD have been implicated in telomere maintenance in other systems, it would be interesting to investigate whether telomere length or sub-telomeric silencing is affected by maintaining strains without NMD for many generations in *C. elegans*.

### The suppression of *sel-12 *by *smg *mutations may involve translational read through

It is surprising that *smg *mutations can sometimes ameliorate *sel-12(PTC) *phenotypes as these transcripts still retain a stop codon and can not encode full length *sel-12 *proteins. Presenilin proteins have eight predicted transmembrane (TM) domains and are normally cleaved in the large cytoplasmic loop between TMs 6 and 7 (for a scheme of the protein see Figure 1B) to generate N and C terminal fragments that remain associated in a protein complex known as the γ-secretase [1]. The γ-secretase also contains three other proteins known as APH-2/Nicastrin, APH-1 and PEN-1. The γ-secretase is an unusual protease complex and all four proteins are required for activity and trafficking to the cell surface of the complex [1]. Partial protein fragments of presenilin proteins are not known to retain protease activity, or to be efficiently assembled in to γ-secretase complexes. This suggests that the suppression of *sel-12 *by *smg *mutations could involve translational read through. We did not directly determine SEL-12 protein levels as we had no antibodies that could detect SEL-12 either in Immunoflorescence or on Western blots. However, our results strongly implicate translational read-through in the mechanism of *sel-12 *suppression by *smg *mutations. The efficiency of read through may be dependent on the type of *sel-12 *allele. The fact that *by146 *ameliorates of the phenotype of both amber stop alleles but not that the *ar171 *opal stop allele is consistent with a read through mechanism specific to *sel-12(amber) *alleles. Proteins involved in NMD have been implicated in modulating read-through of transcripts containing PTCs in yeast [53]. Although all three types of stop codons could be read through in these experiments, the levels of read through of ochre stop mutations was consistently lower than those of amber or opal stop codons. This indicates that the efficiency of read through can be dependent on the type of stop mutation in yeast. In *C. elegans*, amber suppressor tRNA mutations are readily recovered, while opal and ochre specific suppressors are unknown [59]. Thus, in *C. elegans*, informational suppression of amber mutations is more common than that of opal or ochre mutations, consistent with what we see in the *smg; sel-12 *strains.

### The effects of *spr-2 *on *sel-12 *transcript levels do not explain the *spr-2 *phenotype

Curiously, we also found that mutations in *spr-2 *increase the transcript levels of *sel-12(ar171) *and *sel-12(ty11) *but only in the embryo and the L1 larva. The increase in *sel-12(ar171) *or *sel-12(ty11) *transcript levels in the embryo and the L1 stage in a *spr-2 *background is robust, but much weaker than the near restoration of *sel-12 *levels seen in the *smg-2 *background at almost all developmental stages. However, the effects of *spr-2 *on *sel-12 *transcript levels can not explain the suppression of *sel-12 *by *spr-2 *mutations. Firstly, mutations in strong *spr *genes suppress all *sel-12 *alleles that have been tested. For *spr-2*, it was initially shown that *spr-2 *mutations strongly suppress the phenotypes of the *sel-12(ar131) *missense and the *sel-12(ar171) *opal stop mutations [10]. We show that *spr-2 *mutations also strongly suppress the *sel-12(ty11) *amber allele. Secondly, *smg *mutations, which greatly increase *sel-12(PTC) *transcript levels, do not suppress *sel-12 *anywhere near as well as *spr-2 *mutations. This indicates that stabilizing *sel-12(PTC) *transcripts can only weakly ameliorate the phenotype of *sel-12 *mutants. Thus, although the effect of *spr-2 *mutations on *sel-12 *transcript levels might in some cases contribute to the suppression of *sel-12 *phenotypes, it is wholly insufficient to explain the strong *spr-2 *suppressor phenotype. As *spr-2 *mutations do not affect *lin-12 *signaling [10] or *hop-1 *transcript levels (Gontijo and Lakowski unpublished) *spr-2 *mutations suppress *sel-12 *through another, as yet uncharacterized, mechanism that is distinct from both that of NMD and that of the other known *spr *genes.

### How *spr-2 *mutations might affect *sel-12(PTC) *transcript levels

The effect of *spr-2 *mutations on *sel-12(PTC) *transcript levels could be explained if *spr-2 *mutations directly, or indirectly, affect transport out of the nucleus of the mutant *sel-12 *transcripts. Proteins with Nucleosome Assembly Protein (NAP) domains are known to shuttle between the nucleus and the cytoplasm and can function as histone chaperones. The *Aspergillus nidulans *protein most similar to SPR-2, binds to the *A. nidulans *importin α ortholog [60]. Most, if not all, NMD takes place in the cytoplasm and if the export of *sel-12(PTC) *transcripts is affected by *spr-2 *mutations, more of the transcript could be retained in the nucleus and be protected from degradation. In this mechanism, the excess *sel-12 *transcript should have no effect on SEL-12 protein levels as translation also takes place in the cytoplasm. Alternatively, since NMD requires a primary round of translation, *spr-2 *could be necessary for optimal *sel-12 *translation after nuclear export. Reducing translation could thus reduce NMD.

Another possible mechanism could be if *spr-2 *mutations increase *sel-12 *transcription in the embryo and early larva. The human homolog of SPR-2 is known as SET/TAF1beta and has been shown to facilitate transcription [61]. So the effect of *spr-2 *mutations on *sel-12 *transcript levels could be at the level of decreasing transcription of a repressor of *sel-12 *transcription. Paradoxically, SET is also a component of the INHAT (Inhibitor of Histone Acetyltransferase) transcriptional repressor complex, which represses transcription of some loci by blocking the acetylation of histones and thus the activation of transcription [23]. In the absence of this activity, the transcription of a gene could be de-repressed. So *spr-2 *could also be a stage specific repressor of *sel-12 *transcription. However, *spr-2(ar199) *does not obviously affect *sel-12(+) *transcript levels in the embryo and L1 stages (data not shown), suggesting that it is unlikely that *spr-2 *affects *sel-12 *transcription.

Why *spr-2 *mutations have a stage specific effect on *sel-12(PTC) *transcript levels is also unclear. All evidence [10] (and Gontijo and Lakowski unpublished) indicates that *spr-2 *is broadly expressed at all developmental stages. Thus our results suggest that the stage specific effect of *spr-2 *mutations on *sel-12(PTC) *transcript levels might be due to an effect of *spr-2 *on the interaction of *sel-12(PTC) *transcripts and some unknown factor with a stage specific expression pattern. Alternatively, the effect could be due to developmental, or tissue-specific, differences in the effects of *spr-2 *mutations. Determining the role of *spr-2 *in the stage specific regulation of *sel-12(PTC) *transcript levels will require further study.

## Conclusion

Mutations in *spr-2 *increase *sel-12(PTC) *transcript levels in embryos and L1 larvae. However, this effect does not explain the *spr-2 *suppressor phenotype and *spr-2 *does not affect nonsense mediated decay (NMD). The mechanism by which *spr-2 *suppresses *sel-12 *is still unclear but appears to be novel. Mutations in *smg *genes weakly suppress the phenotype of *sel-12 *animals with amber PTC alleles by stabilizing the *sel-12 *transcripts which are otherwise subject to NMD. Some read thorough of *sel-12(amber) *PTCs appears to occur in *smg *backgrounds. Over 100 *spr *mutations have been recovered in screens for suppressors of either the *sel-12(ar171) *opal mutation or the *sel-12(ty11) *amber mutation. However, mutations in *smg *genes are only very infrequently recovered in direct screens for *sel-12 *suppressors. This is probably due to the weakness of their effects on the *sel-12(ty11) *phenotype and the absence of clear effects on the *sel-12(ar171) *phenotype.

## Methods

### Culture conditions, mutations used and strain construction

All worm strains were cultured as previously described [62] and were maintained at 20°C during all experiments unless otherwise stated. The full genotypes of strain used in this study are listed in Additional file 3. The following mutations were used for genetic analysis:

*LGI: smg-2(e2008), dpy-5(e61), smg-1(r861), smg-5(r860), dog-1(gk10) *[63],

unc-54(r293)

LGIII: daf-7(e1372), dpy-1(e1), smg-6(r896), daf-2(e1370) unc-32(e189)

LGIV: smg-3(ma117), smg-7(r1197), let-92(ok1537)

LGV: smg-4(ma116), him-5(e1490)

*LGX: sel-12(ar131 *[4], *ar171 *[4], *by125 *[8], *ok2078, lg1401 *[8], *ty11 *[7]),

unc-1(e538, e719)

*pf126 *is a late Egl and weak *smg*-like Pvl mutation that arose spontaneously in a VC13 *dog-1(gk10) *I mutator strain (Lakowski unpublished).

Many mutations used in this study were provided by the Caenorhabditis Genetics Centre (CGC). The original *spr-2(ar199) *strain was linked to *dpy-20(e1282)*. To study better the phenotypic effects of the *spr-2(ar199) *mutation, we separated *ar199 *from *e1282 *by selecting for Spr non Dpy progeny from *+/spr-2(ar199) dpy-20(e1282); sel-12(ty11) *hermaphrodites. *smg; sel-12 X *strains were constructed by crossing the single mutant strains together and scoring the descendents for the presence of the *sel-12 *Egl phenotype and its amelioration by the *smg *gene. We also scored the mild Pvl phenotype often induced by *smg *mutations to score for their presence in a strain. For most strains, the presence of *smg *mutations was confirmed by the *rpl-12 *test (described below). For *smg-1(by146); sel-12 *strains, the *by146 *deletion was detected by PCR using GN1222 and GN1264 (see Table 1 for primer sequences) on crude worm extracts.

### Isolation of weak *spr *mutations

All *spr *alleles starting with *by *were isolated in screens the lab of Ralf Baumeister as suppressors of the opal stop mutation *sel-12(ar171)*. These screens are described in [11,13] however, only the isolation of strong *spr *mutations was reported in these papers. Additional weaker suppressors were also recovered in these screens but were not characterised at that time. In particular, the *by146 *mutation was recovered in an Ultra Violet light/Tetramethylpsoralen (UV/TMP) screen at 20°C in a *sel-12(ar171) unc-1(e538) *background. *unc-1*, which is closely linked to *sel-12*, was used to facilitate the isolation of suppressor mutations, as Unc-1 animals tend to remain near to the eggs that they have laid. *unc-1 *mutations do not strongly affect the proportion of *sel-12 *animals with an Egl or Pvl phenotype. All alleles starting with *pf*, with the exceptions of *pf167, pf168 *and *pf169*, were isolated in Nematode Genetics Group at the Institut Pasteur as suppressors of the amber stop allele *sel-12(ty11)*. The suppressor screens in the *ty11 *background will be described in greater detail elsewhere. Briefly, the *pf52 *mutation was isolated after Ethylmethanesulfonate (EMS) mutagenesis of a *sel-12(ty11) *strain in essentially the same manner as the screens reported in [13], except that animals were maintained at 25°C instead of the standard 20°C and we deliberately looked for both strong and weaker suppressors in this screen. The *pf167, pf168 *and *pf169 *mutations were isolated in a *sel-12(ar171) unc-1(e538) *background in the same screen as *by146*. These mutations were frozen at that time but were only recently given allele names.

### Staging of worms

To stage worms, plates full of gravid adult hermaphrodites were treated with alkaline hypochlorite solution to kill and dissolve worms [62]. The more resistant eggs were washed three times with M9 buffer and then allowed to hatch overnight in M9 buffer with aeration. Synchronized L1s were then placed onto 9 cm Petri plates to feed and allowed to develop until the desired stage. Staged worms were then washed off the plates, and washed 3 times with M9 before freezing the worm pellet at -80°C until RNA preparation. Where not otherwise specified, L1 corresponds to 5 hours, L2 to 22 hours, L3 to 28 hours, L4 to 44 hours, L4/YA to 50 hours and YA (young adult) to 58 hours of growth. Eggs were not synchronized.

### Isolation of RNA, PCR and Quantitative-PCR

To examine the effect of mutations on transcripts, we extracted total RNA from either mixed stage, or staged worms, depending on the experiment, using TRIZOL reagent following the manufacturer's recommendations (Invitrogen). RNA was treated with Turbo DNAse (Ambion) following their protocol. Reverse transcriptase reactions and quantitative PCR were done using the two-step RT-qPCR Sybr Green low ROX kit by ABgene (reference: AB-1381/a, which is called AB-4116/b since 14/09/2007) according to the manufacturer's recommendations. To synthesize cDNAs we used a 3:1 ratio of random primers and oligo dT primers. Real time PCR reactions were done in 20 μl volumes and run on an Applied Biosystems AB7500 machine in 96 well plates.

For most Q-PCR experiments, the Ct levels of the control genes *ama-1*, *nhx-4 *and *eft-2 *were averaged and this average served as the internal control for the DeltaDeltaCt method to estimate relative fold differences in the target gene RNA levels. The RNA polymerase II large subunit, encoded by the *ama-1 *gene, has often been used as a control in Northern blots including by us [11,13]. *ama-1 *is expressed strongly in all developmental stages, however, we found that it does seem to be even more strongly expressed in embryos than the larval stages (data not shown) consistent with the high level of transcription in embryos and with the fact that *ama-1 *has a strong maternal component. In order to normalize better our studies, we sought additional controls that were broadly expressed housekeeping genes with expression levels both higher and lower than *ama-1*. As a control gene with expression levels lower than *ama-1 *we chose the gene encoding the presumptive housekeeping Na+-H+ exchanger, *nhx-4*, which has been shown to be expressed in all cells at all times examined [64]. As a very highly expressed gene, we chose *eft-2 *[65], a gene that encodes a homolog of translation elongation factor 2 (EF-2), a GTP-binding protein essential for the elongation phase of protein synthesis that is broadly expressed in all stages of development [65]. We determined the absolute transcriptional profiles of these genes (*eft-2*, *ama-1 *and *nhx-4*) under various conditions. The relative expression levels between these three control genes are highly stereotyped during development and reproducible between the strains we are working with (data not shown). Importantly, their expression levels cover 3 log scales and can be very useful for the validation of expression data of genes whose expression vary considerably during development. The names of the products amplified and all PCR primers used in this study are listed in Table 1.

### *rpl-12 *NMD assay

To test the stability of the unproductive splice variant of *rpl-12 *in different strains, we used the protocol of [53]. Briefly, 2 μl of first strand cDNAs were used for PCR with the primers GN1100 and GN1101 to test for *rpl-12*, while amplification with GN701 and GN702 was run in parallel to determine *ama-1 *levels as a control. The PCR program was 5 min at 94°C and then 35 cycles of 94°C for 30 seconds, 58°C for 30 seconds and 72°C for 45 seconds followed by a final step of 7 minutes at 72°C. The products were then migrated on a 2% agarose gel. For *ama-1*, a product of 80 bp is expected, while for *rpl-12*, the reaction can amplify two products of approximately 450 and 550 bp. The 550-bp product retains part of an intron with a PTC codon and is subject to NMD in strains that are proficient in NMD. Strains that are deficient in NMD can be easily identified by the detection of increased amounts of the larger *rpl-12 *product. The original isolates of following alleles were tested: *by106, by111, by115, by118, by120, by121, by122, by123, spr-4(by130), spr-3(by137), by140, by141, by142, by143, by144, by145, by146, pf9, pf14, pf21, pf33, pf48, pf50, pf52, pf53, pf54, pf56, pf57, pf58, pf59, pf60, pf61, pf62, pf63, pf66, pf69, pf70, pf71, pf72, pf73, pf74, pf77, pf119, pf120, pf139, pf145, spr-3(pf154), pf161, pf167, pf168, pf169, spr-3(pf193) *(Figure 4B and data not shown). For all other *spr *alleles the gene affected, and the specific mutation, are known. Two additional mutants were also tested: *pf126 *is a spontaneous mutation that arose in a VC13 *dog-1(gk10) *strain [63] with a mild, *smg*-like protruding vulva (Lakowski unpublished) and *ok1537 *is a mutation in *let-92 *the catalytic subunit of protein phosphatase 2A which has been shown to interact with SMG-5 and SMG-7 to dephosphorylate SMG-2.

### Genetic mapping of *spr-8(pf52)*

To determine linkage for *spr-8(pf52)*, we crossed CB4856 males to *pf52; sel-12(ty11) unc-1(e719) *hermaphrodites, isolated cross progeny and then placed F2 Uncs that were not Egl singly on plates. The Spr phenotype of the strains was confirmed in the F3 generation and lysates from 15-20 of the confirmed broods were pooled for analysis. These lysates, along with N2 and CB4856 control DNA were used to test for the presence of SNPs using a variation of the protocol of [66]. We found strong linkage of *pf52 *to SNP_Y22D7AL[4] on the left arm of chromosome III (see Figure 4A for a genetic map).

To refine the position of *pf52*, we crossed *+/pf52; sel-12(ty11) *males to *daf-7(e1372) dpy-1(e1); sel-12(ty11) *hermaphrodites and isolated cross progeny. We singled all non-Egl F2 progeny from 8 plates and scored their progeny for the various phenotypes. The results of this cross was consistent with *pf52 *being to the right of *dpy-1 *(data not shown). So we crossed *+/pf52 III; sel-12(ty11) X *males to *dpy-1(e1) daf-2(e1370) unc-32(e189)III *hermaphrodites, isolated cross progeny and then isolated Egl F2 progeny. In the F3 we singled Unc non Dpy and Dpy non Unc recombinant progeny from those plates that had some eggs. The final scoring of these recombinants was *dpy-1 ***31 ***spr-8 ***20 ***daf-2 ***41 ***unc-32*, placing *spr-8 *about midway between *dpy-1 *and *daf-2*. We constructed a *dpy-1(e1) spr-8(pf52) unc-32(e189) III; sel-12(ty11) X *strain for further SNP mapping with polymorphisms from the CB4856 strain. However, the results of these experiments were very difficult to interpret due to the weak phenotype of *pf52 *and the presence of polymorphisms that modify the *sel-12 *phenotype in the CB4856 background. However, the results of these mapping experiments were most consistent with *pf52 *mapping between the SNPs *pkP3086 *and SNP_Y71H2B[2] (data not shown).

### Testing the ability of *pf52 *to suppress *unc-54(r193)*

To see if *pf52 *affected NMD more generally, we tested whether *pf52 *could suppress the *smg *suppressible *unc-54(r293) *allele. We crossed *unc-54(r293)/+ *males to *spr-8(pf52) III; sel-12(ty11) unc-1(e719) *hermaphrodites and isolated wild type moving cross progeny. In the F2 generation we randomly picked 19 *Unc-54 *animals individually to plates and then examined the F3 and F4 progeny. All of the progeny in three of 19 F3 broods were nearly wild type, although slightly slow moving and displayed a mild protruding vulva phenotype similar to that seen in all previously identified *smg *mutations. Eight of the 16 remaining broods had some eggs and some animals moving better than *unc-54 *animals usually move. In the F4 generation these eight plates had many wild type moving progeny and 6/8 had some Unc-1 progeny. In the F4 two of the remaining eight broods had no wild type progeny but many animals suppressed for Unc-54 but with Unc-1 movement defects. The remaining six broods were phenotypically Unc-54 in the F3 and F4 generations. These results are consistent with *pf52 *strongly suppressing *unc-54(r293) *but displaying a partial maternal effect.

### Complementation tests for *by146*

We crossed *+/smg-1 *and *+/smg-5 *males to *by146 unc-54(r293) I; sel-12(ar171) unc-1(e538) X *hermaphrodites. We placed 10 cross progeny hermaphrodites for each cross on individual plates as L3/L4 animals. 4/10 cross progeny from the *+/smg-1 *cross had a mild Pvl while none of the cross progeny from the *+/smg-5 *cross did. Three out of the four Pvl animals from the +/*smg-1 *cross segregated no phenotypically Unc-54 progeny in the next generation. The seven remaining *+ *or *smg-1(r861)/by146 unc-54(r293) *plates segregated some phenotypically Unc-54 animals, while all 10 of the *+ *or *smg-5(r860)/by146 unc-54(r293) *plates segregated Unc-54 animals.

### Linkage analysis of *smg-1(by146)*

As we do not know the reason why the CB4856 background modifies the *sel-12 *phenotype, we examined if there were polymorphisms in this strain that could modulate NMD. We crossed CB4856 males to *unc-54(r293) *hermaphrodites, isolated cross progeny and then placed 30 Unc-54 F2 progeny singly on plates. All 30 animals gave rise to strains in which all animals were strongly paralyzed indicating that the CB4856 strain does not contain any polymorphisms that strongly affect NMD. We then crossed CB4856 males to *by146 *hermaphrodites. Using the protruding vulva (Pvl) phenotype of *by146 *we isolated cross progeny and then placed 60 F2 animals with Pvls individually on plates. We examined the F3 generation for a high penetrance of Pvl defect and found 45 strains that we were fairly confident were *smg *based on their highly penetrant mild Pvl phenotype. We made lysates from each of these strains and then pooled 2 μl of each lysate. We then tested these pooled lysates, along with N2 and CB4856 genomic DNA for the presence of SNPs near each of the known *smg *and *smgl *genes (data not shown).

### Reproducibility and statistical analysis

Standard errors of the mean, 95% confidence limits and Student's T-tests were performed using Excel 2003. ANOVA test were calculated using . 95% confidence limits for proportions were calculated in Excel 2003 using formulas 22.26 and 22.27 for the confidence limits for proportions from [67].

For Q-RT-PCR experiments, unless otherwise specified, the normalized average of three biological repeats ± standard error of the mean is shown in the figures. In Figure 1A, we show *sel-12 *transcript levels as determined with three different amplicons (*sel-12-3, sel-12-5 *and *sel-12-*6). In this figure, both *spr-2 *mutations reproducibly increase *sel-12(ar171) *transcript levels. If we average the results for the two cDNAs and consider each amplicon as an independent assessment of *sel-12 *transcript levels, the probabilities that the means are similar are p = 0.047 and p = 0.062 (Student's T-test) for comparison of the means of *ar171 *vs. *ar199; ar171 *and *ar171 *vs. *ar211; ar171 *respectively and at the point of being statistically significant. Additionally, results for the *sel-12-1 *amplicon were very similar (data not shown). For Figure 1D, error bars represent the standard deviation of the mean between at least two independent biological repeats, except for *sel-12(ar171) *where it represents the standard deviation of the mean between two independent qRT-PCR assays from one biological sample (same biological sample as in Figure 1A). In Additional File 2 the results of individual experiments for the determination of egg-laying and brood size are presented. In Figure 6 the pooled results for the individual strains are shown. For the brood size analysis, presenting the results in this manner is justified as no significant differences (p < 0.05) in results for individual strains were detected by T-tests for two repeats, or an ANOVA analysis of variance test for more than two repeats (see Additional file 2, totals).

## List of abbreviations

APP: amyloid precursor protein; Egl: egg-laying defect; NMD: nonsense mediated decay; PTC: premature translation termination codons; Pvl: protruding vulva phenotype; *smg*: suppressor with morphological effects on genitalia; SNP: single nucleotide polymorphism; spr: suppressor of presenilin.

## Competing interests

The authors declare that they have no competing interests.

## Authors' contributions

AMG constructed the first set of *smg sel-12(ty11) *strains, designed and did the Q-PCR studies, analyzed the phenotypes of *smg; sel-12(ty11) *strains, analyzed data and helped write the paper. SA prepared RNA for all uncharacterized *spr *alleles, did the *rpl-12 *test, linkage analysis of *smg-1(by146) *and detection and sequencing of the *by146 *mutation. IR isolated *pf52*, did the complementation tests for *spr-8(pf52) *and the linkage analysis for *pf52*. BL isolated *by146*, did the genetic mapping of *pf52 *and *by146*, constructed and analyzed the *unc-54 smg*, *smg-1(by146); sel-12 *and *smg-6(pf52); sel-12 *double mutant strains and wrote the paper. All authors read and approved the final manuscript.

## Supplementary Material

Additional file 1 **Supplemental figure.** sel-12(by125)*transcripts are subject to NMD and *smg-6(pf52)*partially restores *sel-12(by125)*mRNA levels*. The relative mRNA levels of controls (average between *ama-1, nhx-4 *and *eft-2 *transcript levels) and *sel-12 *transcripts detected with two different primer pairs (for the *sel-12-5 *and *sel-12-6 *amplicons) in mixed stage N2, LA54 *sel-12(by125) *and LA729 *smg-6(pf52); sel-12(by125) *strains. The error bars represent the standard deviation of the mean of two independent qRT-PCR assays from two biological samples per strain. By a one tailed Student's T-test LA729 *smg-6(pf52); sel-12(by125) *transcript levels are significantly higher than LA54 *sel-12(by125) *(P = 0.02). Click here for file

Additional file 2 **Supplemental table.** Table of results of individual phenotype analysis experiments. Click here for file

Additional file 3 **Supplemental table two.** Strains used in this study. The strain names and genotypes of strains used in this study are noted. We also note the original parental strains and the *sel-12 *strains used to construct these strains along with the total number of times the mutation of interest and the *sel-12 *allele have been outcrossed. Click here for file

## References

[B1] Wolfe MS (2006). The gamma-secretase complex: membrane-embedded proteolytic ensemble. Biochemistry.

[B2] L'Hernault SW, Arduengo PM (1992). Mutation of a putative sperm membrane protein in Caenorhabditis elegans prevents sperm differentiation but not its associated meiotic divisions. J Cell Biol.

[B3] Gosney R, Liau WS, Lamunyon CW (2008). A novel function for the presenilin family member spe-4: inhibition of spermatid activation in Caenorhabditis elegans. BMC Dev Biol.

[B4] Levitan D, Greenwald I (1995). Facilitation of lin-12-mediated signalling by sel-12, a Caenorhabditis elegans S182 Alzheimer's disease gene. Nature.

[B5] Li X, Greenwald I (1997). HOP-1, a Caenorhabditis elegans presenilin, appears to be functionally redundant with SEL-12 presenilin and to facilitate LIN-12 and GLP-1 signaling. Proc Natl Acad Sci USA.

[B6] Westlund B, Parry D, Clover R, Basson M, Johnson CD (1999). Reverse genetic analysis of Caenorhabditis elegans presenilins reveals redundant but unequal roles for sel-12 and hop-1 in Notch-pathway signaling. Proc Natl Acad Sci USA.

[B7] Cinar HN, Sweet KL, Hosemann KE, Earley K, Newman AP (2001). The SEL-12 presenilin mediates induction of the Caenorhabditis elegans uterine pi cell fate. Dev Biol.

[B8] Eimer S, Donhauser R, Baumeister R (2002). The Caenorhabditis elegans presenilin sel-12 is required for mesodermal patterning and muscle function. Dev Biol.

[B9] Wu G, Hubbard EJ, Kitajewski JK, Greenwald I (1998). Evidence for functional and physical association between Caenorhabditis elegans SEL-10, a Cdc4p-related protein, and SEL-12 presenilin. Proc Natl Acad Sci USA.

[B10] Wen C, Levitan D, Li X, Greenwald I (2000). spr-2, a suppressor of the egg-laying defect caused by loss of sel-12 presenilin in Caenorhabditis elegans, is a member of the SET protein subfamily. Proc Natl Acad Sci USA.

[B11] Eimer S, Lakowski B, Donhauser R, Baumeister R (2002). Loss of spr-5 bypasses the requirement for the C. elegans presenilin sel-12 by derepressing hop-1. Embo J.

[B12] Jarriault S, Greenwald I (2002). Suppressors of the egg-laying defective phenotype of sel-12 presenilin mutants implicate the CoREST corepressor complex in LIN-12/Notch signaling in C. elegans. Genes Dev.

[B13] Lakowski B, Eimer S, Gobel C, Bottcher A, Wagler B, Baumeister R (2003). Two suppressors of sel-12 encode C2H2 zinc-finger proteins that regulate presenilin transcription in Caenorhabditis elegans. Development.

[B14] Lakowski B, Roelens I, Jacob S (2006). CoREST-like complexes regulate chromatin modification and neuronal gene expression. J Mol Neurosci.

[B15] Hubbard EJ, Wu G, Kitajewski J, Greenwald I (1997). sel-10, a negative regulator of lin-12 activity in Caenorhabditis elegans, encodes a member of the CDC4 family of proteins. Genes Dev.

[B16] Gupta-Rossi N, Le Bail O, Gonen H, Brou C, Logeat F, Six E, Ciechanover A, Israel A (2001). Functional interaction between SEL-10, an F-box protein, and the nuclear form of activated Notch1 receptor. J Biol Chem.

[B17] Wu G, Lyapina S, Das I, Li J, Gurney M, Pauley A, Chui I, Deshaies RJ, Kitajewski J (2001). SEL-10 is an inhibitor of notch signaling that targets notch for ubiquitin-mediated protein degradation. Mol Cell Biol.

[B18] Maruyama S, Hatakeyama S, Nakayama K, Ishida N, Kawakami K (2001). Characterization of a mouse gene (Fbxw6) that encodes a homologue of Caenorhabditis elegans SEL-10. Genomics.

[B19] Oberg C, Li J, Pauley A, Wolf E, Gurney M, Lendahl U (2001). The Notch intracellular domain is ubiquitinated and negatively regulated by the mammalian Sel-10 homolog. J Biol Chem.

[B20] Li J, Pauley AM, Myers RL, Shuang R, Brashler JR, Yan R, Buhl AE, Ruble C, Gurney ME (2002). SEL-10 interacts with presenilin 1, facilitates its ubiquitination, and alters A-beta peptide production. J Neurochem.

[B21] Nagata K, Kawase H, Handa H, Yano K, Yamasaki M, Ishimi Y, Okuda A, Kikuchi A, Matsumoto K (1995). Replication factor encoded by a putative oncogene, set, associated with myeloid leukemogenesis. Proc Natl Acad Sci USA.

[B22] Li M, Makkinje A, Damuni Z (1996). The myeloid leukemia-associated protein SET is a potent inhibitor of protein phosphatase 2A. J Biol Chem.

[B23] Seo SB, McNamara P, Heo S, Turner A, Lane WS, Chakravarti D (2001). Regulation of histone acetylation and transcription by INHAT, a human cellular complex containing the set oncoprotein. Cell.

[B24] Cervoni N, Detich N, Seo SB, Chakravarti D, Szyf M (2002). The oncoprotein Set/TAF-1beta, an inhibitor of histone acetyltransferase, inhibits active demethylation of DNA, integrating DNA methylation and transcriptional silencing. J Biol Chem.

[B25] ten Klooster JP, Leeuwen I, Scheres N, Anthony EC, Hordijk PL (2007). Rac1-induced cell migration requires membrane recruitment of the nuclear oncogene SET. Embo J.

[B26] Canela N, Rodriguez-Vilarrupla A, Estanyol JM, Diaz C, Pujol MJ, Agell N, Bachs O (2003). The SET protein regulates G2/M transition by modulating cyclin B-cyclin-dependent kinase 1 activity. J Biol Chem.

[B27] Baker KE, Parker R (2004). Nonsense-mediated mRNA decay: terminating erroneous gene expression. Curr Opin Cell Biol.

[B28] Conti E, Izaurralde E (2005). Nonsense-mediated mRNA decay: molecular insights and mechanistic variations across species. Curr Opin Cell Biol.

[B29] Lejeune F, Maquat LE (2005). Mechanistic links between nonsense-mediated mRNA decay and pre-mRNA splicing in mammalian cells. Curr Opin Cell Biol.

[B30] Hodgkin J, Papp A, Pulak R, Ambros V, Anderson P (1989). A new kind of informational suppression in the nematode Caenorhabditis elegans. Genetics.

[B31] Pulak R, Anderson P (1993). mRNA surveillance by the Caenorhabditis elegans smg genes. Genes Dev.

[B32] Cali BM, Kuchma SL, Latham J, Anderson P (1999). smg-7 is required for mRNA surveillance in Caenorhabditis elegans. Genetics.

[B33] Longman D, Plasterk RH, Johnstone IL, Caceres JF (2007). Mechanistic insights and identification of two novel factors in the C. elegans NMD pathway. Genes Dev.

[B34] Leeds P, Peltz SW, Jacobson A, Culbertson MR (1991). The product of the yeast UPF1 gene is required for rapid turnover of mRNAs containing a premature translational termination codon. Genes Dev.

[B35] Leeds P, Wood JM, Lee BS, Culbertson MR (1992). Gene products that promote mRNA turnover in Saccharomyces cerevisiae. Mol Cell Biol.

[B36] Page MF, Carr B, Anders KR, Grimson A, Anderson P (1999). SMG-2 is a phosphorylated protein required for mRNA surveillance in Caenorhabditis elegans and related to Upf1p of yeast. Mol Cell Biol.

[B37] Johns L, Grimson A, Kuchma SL, Newman CL, Anderson P (2007). Caenorhabditis elegans SMG-2 selectively marks mRNAs containing premature translation termination codons. Mol Cell Biol.

[B38] Cui Y, Hagan KW, Zhang S, Peltz SW (1995). Identification and characterization of genes that are required for the accelerated degradation of mRNAs containing a premature translational termination codon. Genes Dev.

[B39] Lee BS, Culbertson MR (1995). Identification of an additional gene required for eukaryotic nonsense mRNA turnover. Proc Natl Acad Sci USA.

[B40] Grimson A, O'Connor S, Newman CL, Anderson P (2004). SMG-1 is a phosphatidylinositol kinase-related protein kinase required for nonsense-mediated mRNA Decay in Caenorhabditis elegans. Mol Cell Biol.

[B41] Fukuhara N, Ebert J, Unterholzner L, Lindner D, Izaurralde E, Conti E (2005). SMG7 is a 14-3-3-like adaptor in the nonsense-mediated mRNA decay pathway. Mol Cell.

[B42] Anders KR, Grimson A, Anderson P (2003). SMG-5, required for C. elegans nonsense-mediated mRNA decay, associates with SMG-2 and protein phosphatase 2A. Embo J.

[B43] Chiu SY, Serin G, Ohara O, Maquat LE (2003). Characterization of human Smg5/7a: a protein with similarities to Caenorhabditis elegans SMG5 and SMG7 that functions in the dephosphorylation of Upf1. Rna.

[B44] Ohnishi T, Yamashita A, Kashima I, Schell T, Anders KR, Grimson A, Hachiya T, Hentze MW, Anderson P, Ohno S (2003). Phosphorylation of hUPF1 induces formation of mRNA surveillance complexes containing hSMG-5 and hSMG-7. Mol Cell.

[B45] Glavan F, Behm-Ansmant I, Izaurralde E, Conti E (2006). Structures of the PIN domains of SMG6 and SMG5 reveal a nuclease within the mRNA surveillance complex. Embo J.

[B46] Gatfield D, Unterholzner L, Ciccarelli FD, Bork P, Izaurralde E (2003). Nonsense-mediated mRNA decay in Drosophila: at the intersection of the yeast and mammalian pathways. Embo J.

[B47] Reichenbach P, Hoss M, Azzalin CM, Nabholz M, Bucher P, Lingner J (2003). A human homolog of yeast Est1 associates with telomerase and uncaps chromosome ends when overexpressed. Curr Biol.

[B48] Snow BE, Erdmann N, Cruickshank J, Goldman H, Gill RM, Robinson MO, Harrington L (2003). Functional conservation of the telomerase protein Est1p in humans. Curr Biol.

[B49] Luke B, Azzalin CM, Hug N, Deplazes A, Peter M, Lingner J (2007). Saccharomyces cerevisiae Ebs1p is a putative ortholog of human Smg7 and promotes nonsense-mediated mRNA decay. Nucleic Acids Res.

[B50] Lew JE, Enomoto S, Berman J (1998). Telomere length regulation and telomeric chromatin require the nonsense-mediated mRNA decay pathway. Mol Cell Biol.

[B51] Azzalin CM, Reichenbach P, Khoriauli L, Giulotto E, Lingner J (2007). Telomeric repeat containing RNA and RNA surveillance factors at mammalian chromosome ends. Science.

[B52] Chang YF, Imam JS, Wilkinson MF (2007). The nonsense-mediated decay RNA surveillance pathway. Annu Rev Biochem.

[B53] Mitrovich QM, Anderson P (2000). Unproductively spliced ribosomal protein mRNAs are natural targets of mRNA surveillance in C. elegans. Genes Dev.

[B54] Cali BM, Anderson P (1998). mRNA surveillance mitigates genetic dominance in Caenorhabditis elegans. Mol Gen Genet.

[B55] Domeier ME, Morse DP, Knight SW, Portereiko M, Bass BL, Mango SE (2000). A link between RNA interference and nonsense-mediated decay in Caenorhabditis elegans. Science.

[B56] Levitan D, Doyle TG, Brousseau D, Lee MK, Thinakaran G, Slunt HH, Sisodia SS, Greenwald I (1996). Assessment of normal and mutant human presenilin function in Caenorhabditis elegans. Proc Natl Acad Sci USA.

[B57] Huntzinger E, Kashima I, Fauser M, Sauliere J, Izaurralde E (2008). SMG6 is the catalytic endonuclease that cleaves mRNAs containing nonsense codons in metazoan. Rna.

[B58] Eberle AB, Lykke-Andersen S, Muhlemann O, Jensen TH (2009). SMG6 promotes endonucleolytic cleavage of nonsense mRNA in human cells. Nat Struct Mol Biol.

[B59] Hodgkin J (2005). Genetic suppression. WormBook.

[B60] Araujo-Bazan L, Fernandez-Martinez J, Rios VM, Etxebeste O, Albar JP, Penalva MA, Espeso EA (2008). NapA and NapB are the Aspergillus nidulans Nap/SET family members and NapB is a nuclear protein specifically interacting with importin alpha. Fungal Genet Biol.

[B61] Okuwaki M, Nagata K (1998). Template activating factor-I remodels the chromatin structure and stimulates transcription from the chromatin template. J Biol Chem.

[B62] Sulston J, Hodgkin J, Wood W (1988). Methods. The Nematode Caenorhabditis elegans.

[B63] Cheung I, Schertzer M, Rose A, Lansdorp PM (2002). Disruption of dog-1 in Caenorhabditis elegans triggers deletions upstream of guanine-rich DNA. Nat Genet.

[B64] Nehrke K, Melvin JE (2002). The NHX family of Na+-H+ exchangers in Caenorhabditis elegans. J Biol Chem.

[B65] Ofulue EN, Candido EP (1991). Molecular cloning and characterization of the Caenorhabditis elegans elongation factor 2 gene (eft-2). DNA Cell Biol.

[B66] Wicks SR, de Vries CJ, van Luenen HGAM, Plasterk RHA (2000). CHE-3, a Cytosolic Dynein Heavy Chain, Is Required for Sensory Cilia Structure and Function in Caenorhabditis elegans. Deveopmental Biology.

[B67] Zar JH (1984). Biostatistical Analysis.

