# Supplementary figures and images for: Mutations in genes involved in nonsense mediated decay ameliorate the phenotype of sel-12 mutants with amber stop mutations in Caenorhabditis elegans
Source: BMC Genet. 2009 Mar 20;10:14. doi: 10.1186/1471-2156-10-14 (PMC2678165; doi:10.1186/1471-2156-10-14)

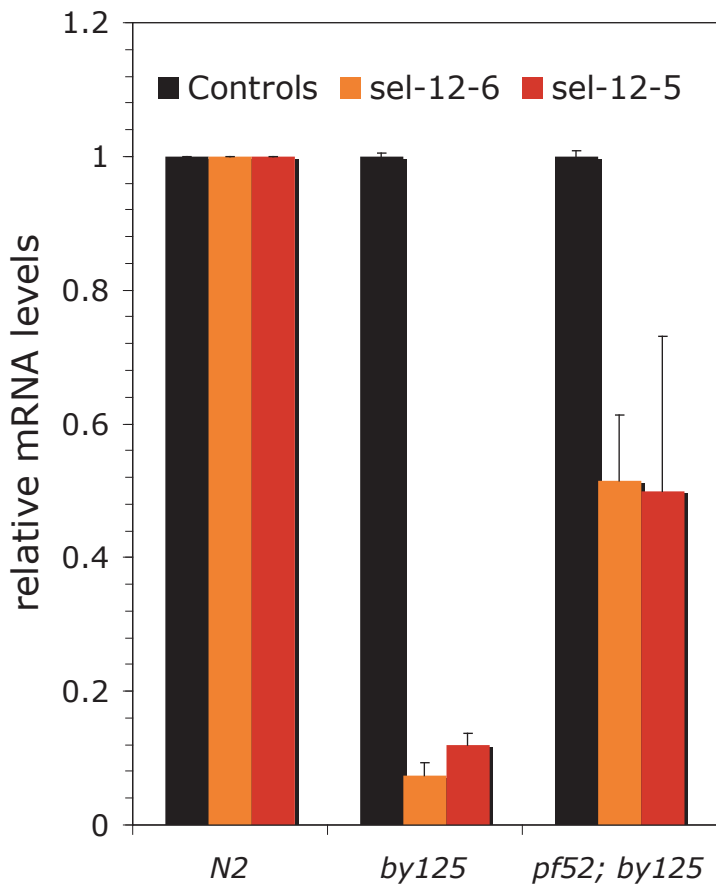

Supplement: Additional file 1 — Supplemental figure. sel-12(by125)transcripts are subject to NMD and smg-6(pf52)partially restores sel-12(by125)mRNA levels. The relative mRNA levels of controls (average between ama-1, nhx-4 and eft-2 transcript levels) and sel-12 transcripts detected with two different primer pairs (for the sel-12-5 and sel-12-6 amplicons) in mixed stage N2, LA54 sel-12(by125) and LA729 smg-6(pf52); sel-12(by125) strains. The error bars represent the standard deviation of the mean of two independent qRT-PCR assays from two biological samples per strain. By a one tailed Student's T-test LA729 smg-6(pf52); sel-12(by125) transcript levels are significantly higher than LA54 sel-12(by125) (P = 0.02). [file 1471-2156-10-14-S1.pdf]
