# Supplementary material for: Mutations in genes involved in nonsense mediated decay ameliorate the phenotype of sel-12 mutants with amber stop mutations in Caenorhabditis elegans
Source: BMC Genet. 2009 Mar 20;10:14. doi: 10.1186/1471-2156-10-14 (PMC2678165; doi:10.1186/1471-2156-10-14)
Supplement: Additional file 2 — Supplemental table. Table of results of individual phenotype analysis experiments. [file 1471-2156-10-14-S2.doc]

Egg laying and bood size experiments.

| strain  name | genotype | brood  size | 95% | n1 | P= | egg  laying | n2 |
| --- | --- | --- | --- | --- | --- | --- | --- |
|  |  |  |  |  |  |  |  |
| **expt. 1** |  |  |  |  | vs. *ty11* |  |  |
| LA242 | *sel-12(ty11)* | 73.6 | 16.4 | 14 |  | 3 | 37 |
| LA259 | *spr-2(ar199); sel-12( ty11)* | 183.1 | 12.9 | 18 |  | 20 | 20 |
| LA905 | *spr-2(ar199)* | 140.2 | 12.7 | 19 |  |  |  |
| LA613 | *smg-1(r861); sel-12(ty11)* | 72.8 | 12.1 | 15 | 0.941 | 6 | 15 |
|  | *smg-2(e2008); sel-12(ty11)* | 84.4 | 18.7 | 16 | 0.426 | 14 | 20 |
| LA771 | *smg-3(ma117); sel-12(ty11)* | 51.2 | 13.3 | 10 | 0.074 |  |  |
| LA623 | *smg-4(ma116); sel-12(ty11)* | 68.7 | 15.0 | 18 | 0.680 |  |  |
| LA617 | *smg-5(r860); sel-12(ty11)* | 85.8 | 20.7 | 14 | 0.397 |  |  |
| LA622 | *smg-6(r896); sel-12(ty11)* | 66.1 | 18.6 | 14 | 0.570 |  |  |
|  |  |  |  |  |  |  |  |
| **expt.2** |  |  |  |  | vs. *ar131* |  |  |
| LA728 | *sel-12(ar131)* | 109.2 | 20.4 | 25 |  | 14 | 25 |
| LA726 | *smg-2(e2008); sel-12(ar131)* | 76.7 | 19.1 | 22 | 0.029 | 11 | 22 |
| LA725 | *smg-2(e2008); sel-12(ar131)* | 100.3 | 20.8 | 22 | 0.554 | 10 | 22 |
| LA722 | *smg-5(r860); sel-12(ar131)* | 75.5 | 18.6 | 23 | 0.022 | 17 | 23 |
| LA723 | *smg-5(r860); sel-12(ar131)* | 100.6 | 20.5 | 24 | 0.566 | 18 | 24 |
| LA727 | *smg-6(pf52); sel-12(ar131)* | 97.1 | 15.1 | 23 | 0.364 | 17 | 23 |
|  |  |  |  |  |  |  |  |
| **expt.3** |  |  |  |  | vs. appropriate  *sel-12* allele |  |  |
| N2 | *+* | 312.3 | 30.9 | 20 |  | 20 | 20 |
| LA259 | *sel-12(ty11)* | 83.7 | 13.3 | 20 |  | 1 | 20 |
| LA54 | *sel-12(by125)* | 73.7 | 11.1 | 20 |  | 1 | 20 |
| LA729 | *smg-6(pf52); sel-12(by125)* | 132.7 | 25.0 | 19 | 0.0001 | 12 | 18 |
| LA423 | *smg-6(pf52); sel-12(ty11)* | 144.7 | 15.1 | 18 | 8X10-7 | 15 | 17 |
|  |  |  |  |  |  |  |  |
| **expt. 4** |  |  |  |  |  |  |  |
| N2 | *+* | 327.9 | 19.1 | 19 |  | 19 | 19 |
| LA259 | *sel-12(ty11)* | 94.1 | 17.8 | 18 |  | 2 | 18 |
|  |  |  |  |  |  |  |  |
| **expt.5** |  |  |  |  | vs. appropriate  *sel-12* allele |  |  |
| LA920 | *smg-1(by146)* | 268.6 | 13.9 | 20 |  | 20 | 20 |
| BR1253 | *sel-12(ar171)* | 91.6 | 17.4 | 18 |  | 1 | 18 |
| LA54 | *sel-12(by125)* | 95.1 | 14.5 | 19 |  | 1 | 19 |
| LA913 | *sel-12(ok2058)* | 82.2 | 15.9 | 19 |  | 2 | 19 |
| LA242 | *sel-12(ty11)* | 81.3 | 16.1 | 20 |  | 1 | 20 |
| LA911 | *smg-1(by146) ; sel-12(ar171)* | 76.4 | 16.6 | 16 | 0.175 | 0 | 16 |
| LA910 | *smg-1(by146) ; sel-12(by125)* | 130.3 | 21.1 | 19 | 0.014 | 16 | 19 |
| LA912 | *smg-1(by146) ; sel-12(ok2058)* | 91.3 | 16.6 | 19 | 0.540 | 4 | 19 |
| LA909 | *smg-1(by146) ; sel-12(ty11)* | 110.5 | 20.9 | 17 | 0.044 | 11 | 17 |
| strain  name | genotype | brood  size | 95% | n1 | P= | egg  laying | n2 |
|  |  |  |  |  |  |  |  |
| **expt.6** |  |  |  |  | vs. appropriate  *sel-12* allele |  |  |
| N2 | *+* | 345.5 | 14.1 | 20 |  | 20 | 20 |
| LA920 | *smg-1(by146)* | 263.5 | 16.2 | 40 |  | 40 | 40 |
| LA899 | *sel-12(ar131)* | 138.4 | 19.3 | 40 |  | 27 | 40 |
| BR1253 | *sel-12(ar171)* | 88.3 | 15.6 | 20 |  | 2 | 20 |
| LA54 | *sel-12(by125)* | 77.1 | 14.6 | 20 |  | 2 | 20 |
| LA913 | *sel-12(ok2058)* | 95.3 | 14.4 | 19 |  | 4 | 19 |
| LA242 | *sel-12(ty11)* | 67.4 | 10.5 | 20 |  | 1 | 20 |
| LA911 | *smg-1(by146) ; sel-12(ar171)* | 97.9 | 22.8 | 17 | 0.302 | 6 | 17 |
| LA910 | *smg-1(by146) ; sel-12(by125)* | 109.3 | 20.9 | 19 | 0.010 | 9 | 20 |
| LA912 | *smg-1(by146) ; sel-12(ok2058)* | 85.3 | 13.6 | 19 | 0.250 | 2 | 19 |
| LA909 | *smg-1(by146) ; sel-12(ty11)* | 97.1 | 22.4 | 17 | 0.043 | 10 | 17 |
| BR2097 | *smg-1(by146) ; sel-12(ar171) unc-1(e538)* | 39.9 | 7.5 | 31 | 2X10-7 | 4 | 40 |
|  |  |  |  |  |  |  |  |
| **totals** |  |  |  |  | pooling samples |  |  |
| N2 | + | 328.6 | 13.3 | 59 | 0.130 A | 59 | 59 |
| LA920 | *smg-1(by146)* | 265.2 | 11.7 | 60 | 0.900 A | 60 | 60 |
|  | *sel-12(ar131)* | 127.2 | 14.6 | 65 | 0.054 | 41 | 65 |
|  | *smg-2(e2008); sel-12(ar131)* | 88.5 | 14.4 | 44 | 0.110 | 21 | 44 |
|  | *smg-5(r860); sel-12(ar131)* | 88.3 | 14.2 | 47 | 0.084 | 35 | 47 |
| LA727 | *smg-6(pf52); sel-12(ar131)* | 97.1 | 15.1 | 23 |  | 17 | 23 |
| BR1253 | *sel-12(ar171)* | 89.9 | 11.5 | 38 | 0.780 | 3 | 38 |
| LA54 | *sel-12(by125)* | 81.7 | 8.0 | 59 | 0.073 A | 4 | 59 |
| LA913 | *sel-12(ok2058)* | 88.7 | 10.8 | 38 | 0.237 | 6 | 38 |
| LA242 | *sel-12(ty11)* | 81.3 | 7.4 | 78 | 0.190 A | 5 | 78 |
| LA911 | *smg-1(by146) ; sel-12(ar171)* | 87.5 | 14.5 | 33 | 0.148 | 6 | 33 |
| LA910 | *smg-1(by146) ; sel-12(by125)* | 119.8 | 15.0 | 38 | 0.174 | 25 | 39 |
| LA912 | *smg-1(by146) ; sel-12(ok2058)* | 88.8 | 10.5 | 38 | 0.519 | 6 | 38 |
| LA909 | *smg-1(by146) ; sel-12(ty11)* | 104.0 | 15.2 | 33 | 0.397 | 21 | 34 |
| BR2097 | *smg-1(by146) ; sel-12(ar171) unc-1(e538)* | 39.9 | 7.5 | 31 |  | 4 | 40 |
| LA729 | *smg-6(pf52); sel-12(by125)* | 132.7 | 25.0 | 19 |  | 12 | 18 |
| LA423 | *smg-6(pf52); sel-12(ty11)* | 144.7 | 15.1 | 17 |  | 15 | 17 |
|  |  |  |  |  |  |  |  |
|  | *sel-12(0)* | 84.3 | 4.5 | 213 | 0.120 A | 18 | 213 |
|  | *pf52; sel-12* amber | 138.4 | 14.9 | 36 | 0.439 | 27 | 35 |
|  | *by146; sel-12* amber | 112.4 | 10.8 | 71 | 0.200 A | 46 | 73 |
|  | *by146; sel-12* non amber null | 88.2 | 8.7 | 71 | 0.390 A | 12 | 71 |

95% is the 95% confidence limits for the mean. n1 is the sample size for brood size and n2 is the sample size for egg laying. These are often, but not always the same. For each experiment some animals were excluded from the analysis of brood size, egg laying or both due to one of several reasons including: sterility, death from extrusion of the intestine or gonad from the vulval opening or death due to desiccation on the walls of the Petri plate. Animals were randomly picked as L4 larvae and were repeatedly scored over the next two days for the presence of eggs on the plates. Progeny of these animals were counted as L3-young adults.

P= the probability that the average brood size is the same as an appropriate control. For experiments 1-6 a two-tailed Student’s T-test was done assuming equal variances using Excel 2003 as we were comparing only two strains or repeats. P values that are less than 0.05 are highlighted in yellow. For Expt. 1 *smg; sel-12(ty11)* strains were compared with LA242 *sel-12(ty11)*. For Expt. 2 *smg; sel-12(ar131)* strains were compared with LA728 *sel-12(ar131)*. For Expts.3, 5 and 6, *smg; sel-12* strains were compared with the appropriate *sel-12* allele tested in that experiment. For the totals, P= the probability that all repeats of that genotype have the same brood size. A two-tailed Student’s T-test was done where only two samples were compared, however when we compared multiple samples, an ANOVA test was used (<http://www.physics.csbsju.edu/stats/anova.html>). If this was the case this is indicated by the presence of an A after the P value. For the totals, where no P value is given, only one repeat was done. Where no strain name is indicated, the P value is for the comparison of two different strains with the same known genotype. However, these strains may have different background mutations or other differences. These strains may actually behave differently; however if they do, the differences are not large enough to be statistically significant with the samples sizes used.
